# *Fasciola hepatica* in alpine dairy farming: prevalence trends, risk factors and associations with *Salmonella* Dublin seropositivity

**DOI:** 10.1186/s12917-026-05383-1

**Published:** 2026-02-28

**Authors:** Severin Schuler, Kerstin Hofer, Thomas Wittek, Monika Matt, Nicola Palmieri, Annette Nigsch, Alexander Tichy, Anja Joachim, Michael Dünser, Christian Mader, Barbara Hinney

**Affiliations:** 1 Department of Biological Sciences and Pathobiology, Institute of Parasitology, University of Veterinary Medicine, Vienna, Austria; 2 Institute for Veterinary Disease Control, Austrian Agency for Health and Food Safety (AGES), Linz, Austria; 3 Clinical Department for Farm Animals and Food System Science, Clinical Centre for Ruminant and Camelid Medicine, University of Veterinary Medicine, Vienna, Austria; 4 Data, Statistics and Risk Assessment, Austrian Agency for Health and Food Safety (AGES), Innsbruck, Austria; 5 Department for Farm Animals and Veterinary Public Health, Clinical Centre for Population Medicine in Fish, Pig and Poultry, University of Veterinary Medicine, Vienna, Austria; 6 Institute for Veterinary Disease Control, Austrian Agency for Health and Food Safety (AGES), Innsbruck, Austria; 7 Department for Biomedical Sciences, Bioinformatics and Biostatistics Platform, University of Veterinary Medicine, Vienna, Austria; 8 Animal Health Service Tyrol (T-TGD), Innsbruck, Austria

**Keywords:** *Fasciola hepatica*; Alpine grazing, dairy breed, Bulk tank milk, production, *Salmonella* Dublin, Risk factors, Epidemiology

## Abstract

*Fasciola hepatica* impairs animal health and productivity in ruminants and has been associated with *Salmonella* Dublin infection, a zoonotic pathogen in cattle that causes diarrhea, abortions, and pneumonia. This work reassessed *F. hepatica* seroprevalence in Tyrolean dairy herds and investigated associations with alpine pasturing, the breed, *S.* Dublin occurrence and milk performance. In late autumn/early winter 2023, bulk tank milk samples from 3645 farms were tested using a commercial ELISA test (IDEXX Fasciolosis Verification Test, Montpellier, France). Binary logistic regression and linear mixed models were used to evaluate risk factors and production effects. Overall, 86.1% of herds were positive for *Fasciola* antibodies - an increase of 13.1% compared to 2005. Alpine pasturing was a significant risk factor (odds ratio [OR] 4.7), and *S.* Dublin-positive farms had higher odds of *Fasciola*-infection (OR = 9.0). In non-alpine grazing herds, all models showed that *Fasciola*-infection was significantly associated with reduced milk yield, while no such associations were observed in the population with alpine pasture management. However, breed-specific analysis among alpine-pastured cows showed significant negative associations on milk yield in Simmental (*p* = 0.041) and Brown Swiss (*p* = 0.039). These results reveal an increasing *F. hepatica* prevalence in Tyrol and highlight alpine pasturing as a major risk factor. The observed production losses and the strong link with *S.* Dublin emphasize the parasite`s broader economic and public health importance for dairy farms in the European Alps. From a practical perspective, there is a clear need for region-specific *F. hepatica* control strategies in alpine dairy systems, with particular focus on pasture management and herd-level surveillance.

**Supplementary Information:**

The online version contains supplementary material available at 10.1186/s12917-026-05383-1.

## Introduction

The common liver fluke *Fasciola hepatica* is an endoparasite of major importance in grazing ruminant livestock worldwide [1]. While infections in small ruminants regularly lead to serious animal health problems [2], liver fluke infections in cattle typically present as chronic and without clinical signs [3]. However, *Fasciola hepatica* infection is associated with chronic hepatic inflammation, bile duct hyperplasia and progressive tissue damage, and the infection in cattle frequently leads to significant production losses [4–8].

For completing its life cycle, *F. hepatica* requires a lymnaeid freshwater snail as intermediate host [9]. Parasite occurrence is thus largely dependent on the snail’s environmental preferences for temporary and permanent water reservoirs [10], and mild winters (often seen as a sign of climate change) with high amounts of rainfall combined with extended grazing periods have been associated with increasing liver fluke prevalence in Europe at the herd as well as the individual animal level [10–15]. Additionally, control of *F. hepatica* is complicated by the increasing occurrence of anthelminthic resistance as well as the environmental toxicity of flukicidal compounds [16, 17].

*Fasciola hepatica* is also considered a zoonotic agent [18–20], although reported human cases are rare in central Europe [21]. Of additional animal and public health interest, *F. hepatica* has been suggested as a risk factor for infection with the zoonotic *Salmonella enterica* subspecies *enterica* serovar Dublin (*S.* Dublin) in cattle [22]. This Gram-negative bacterium can cause severe disease in humans, leading to hospitalization and mortality [23, 24]. Several clinical manifestations are also observed in cattle, e.g. watery to hemorrhagic diarrhea and abortions in adults or lethargy and pneumonia in young calves [25–29]. The epidemiology of *F. hepatica* is therefore also important in the One Health context [30], and monitoring its occurrence and effects contributes to the understanding and control of its spread. Despite the importance of alpine dairy farming systems, there is a lack of comprehensive and up-to-date data on the epidemiology, production impact and management-related risk factors for *F. hepatica* infections under alpine farming conditions.

In Austria, dairy farming is the most relevant agricultural sector [31]. Among the special features of Austrian dairy farming are the small farm size (average herd size: 19 cows), the high reliance on dual-purpose breeds in ~ 90% of the herds and alpine farming including transhumance [32–34]. The traditional alpine cattle production system is characterized by animals being kept in the valley during winter and moved to high alpine areas in summer [35]. Alpine cattle farming plays a vital role in preserving the unique cultural landscape of the Alps, keeping open areas free from forest overgrowth and enhancing biodiversity [35, 36]. Furthermore, it enables the use of alpine grasslands that would otherwise remain inaccessible or unused [37]. Nevertheless, a challenge inherent to cattle farming in elevated areas is the frequent identification of alpine regions as highly endemic for *Fasciola* [38–40]. A study in the Austrian federal state of Tyrol in 2005 revealed a high herd-level prevalence of *F. hepatica* on dairy farms, averaging 73%, including some high-prevalence zones with up to 97% positive herds [39].

Most studies on the association between fluke infection and production parameters have been conducted in Holstein dairy cows [4–6]. However, since there are indications that dual-purpose breeds may not show comparable production losses [41], investigating this association in a region with predominantly dual-purpose breeds could help to expand on previous findings.

For targeted and sustainable control of *F. hepatica*, it was recently proposed that the development of reliable predictive models requires empirical data at the local scale across different geographic regions [42], because the occurrence and abundance of this parasite are influenced not only by climatic conditions, but also by factors such as farm management practices. Long-term monitoring at the local level is therefore essential to validate and improve the models. However, to date, only few representative longitudinal studies exist [43, 44] and none have been conducted in alpine regions. The investigations on *F. hepatica* occurrence and associated risk factors in Tyrol can constitute a reference for dairy farming in elevated areas for the validation of climate models and effective parasite control strategies.

Since 2005, temperature and precipitation in Tyrol are constantly increasing [45], suggesting a probable increase in the incidence of *F. hepatica*. To show the long-term effects of climate change, re-examination of animals over time could reveal changes in parasite distribution and/or prevalence.

Correlating the results from *F. hepatica*-positive farms with *S.* Dublin seroprevalence could confirm the previously reported association between the two pathogens in the investigated herds and regions. An earlier field study suggested this link based on self-reported *F. hepatica* infection status in cattle herds, rather than on laboratory-confirmed diagnosis [22].

The aims of the present study were to assess the current prevalence of *F. hepatica* in dairy herds based on bulk tank milk (BTM) samples from all milk-supplying farms in Tyrol and to compare the results with those obtained in 2005 [39]. With these data we wanted to evaluate temporal trends and establish a base line for future monitoring under changing environmental and management conditions. Furthermore, we aimed to evaluate the relationship between infection intensity and farm-management practices as well as breed in alpine cattle systems. In addition, we investigated potential associations between *F. hepatica* seropositivity and production parameters especially with regard to the specific farm management practices in alpine regions with a predominance of Simmental cattle, followed by Brown Swiss. We also wanted to verify the proposed link between *F. hepatica* and *S.* Dublin under field conditions using laboratory-confirmed data from a large number of dairy herds.

Overall, the aim of this study was thus to characterize the current epidemiology, management- and breed-associated risk factors, and production relevance of *F. hepatica* in alpine dairy farming systems in Tyrol. Based on these objectives, the present study tested the following hypotheses: 1., the prevalence of *F. hepatica* has increased since 2005; 2., alpine and communal alpine pasturing are associated with higher antibody titers; 3., higher *F. hepatica* seropositivity is associated with reduced production performance, but that this association is weaker in Simmental cattle compared to other breeds; 4., herds positive for *F. hepatica* are more likely to also be positive for *Salmonella* Dublin.

## Materials and methods

### Study design and study area

This study represents a cross-sectional analysis linking serological, production, and management data at the farm level. The study was based on milk samples collected during a national surveillance program. As all data were anonymized and analyzed in aggregated form, neither ethical approval nor owner consent was required.

The study was conducted in October and November 2023 in Tyrol, Austria. The federal state of Tyrol is characterized by Alpine landscapes in a continental climate with an average annual temperature of 4.5 °C and a precipitation of 1612 mm in 2023 [45]. A total of 4018 dairy farms were recorded by the Tyrolian state government in 2022 [46]. In 2020, the total number of alpine pastures in Tyrol was 2,073, and thus the highest number in Austria. Each year, more than 106,000 cattle, including 31,000 dairy cows, graze on alpine pastures in Tyrol, highlighting the exceptional importance of Alpine grazing systems in this region within the European Alps [47]. Alpine pasturing depends on weather and other management conditions and commonly occurs between May and September. According to the national register 94% of Tyrolian dairy herds had at least a three months grazing period (with or without alpine pasture) [47]. The altitude of alpine pasturing varies considerably according to regional differences, ranging from approximately 800 to 2500 m above sea level, with the western part characterized by higher altitudes than the east.

### Study population and collection of production data and grazing classification

The present study comprised 3645 dairy herds (> 90% of all officially registered dairy farms in Tyrol) that were contracted to supply milk to different milk facilities. The Milk Recording Service Tyrol (“Landeskontrollverband Tirol”) supplied farm and milk production data from the year 2023 at herd level, including the number of dairy cows, the dominating breed (reflecting the predominant breed composition in herds with both purebred and crossbred animals) and the mean calving interval. Further information was supplied, including milk production data (milk kg, fat% and protein%) from 3148 herds. In addition, the Ministry of Agriculture and Forestry provided pasture management data from 2805 Tyrolean dairy farms, and the information included whether farms applied organic or conventional production methods, whether cattle were pastured on alpine sites (at least three months/year), and if so, whether this involved communal grazing (a practice where animals from different farms share one alpine pasture). Other information included whether individual farms pastured dairy cows and how many cows of the farms were registered for alpine grazing.

Besides the information on alpine pasturing as described above data on pasture management were also available from the agri-environmental program “ÖPUL-Weide”, which included farms providing pasture access (lowland pasture) for at least four months per year to one or more animal categories. Thus, certain farms registered in ÖPUL-Weide only grazed exclusively young stock or non-lactating cows, while lactating dairy cows remained confined indoors. Consequently, it was impossible to determine with certainty which farms registered in this program without alpine pasture actually grazed their dairy cows. Farms not participating in either the alpine grazing program (< 3 months) or ÖPUL-Weide (< 4 months) were assumed to provide no or only limited grazing access to their animals.

No information on fasciolicidal treatments was available for the studied farms.

An overview of the number of farms per category is provided in the supplementary information (Additional file 1). This study followed investigations performed by Matt et al. 2007 [39] and Hofer et al. 2024 [48] using BTM samples.

### Collection of BTM samples

As part of the official annual Bovine Viral Diarrhea (BVD) surveillance program, BTM samples from all farms that supply dairy plants in Tyrol (3645) were collected once and analyzed for *F. hepatica* specific antibodies (ABs) using a commercial ELISA kit (IDEXX Fasciolosis Verification Test, Montpellier, France). This ELISA is based on the detection of “f2” antigen, a purified fraction of excretory-secretory (ES) antigens, and is highly specific for *F. hepatica* [49]. In 2022, an analysis was conducted by Hofer et al. 2024 [48] testing for *S.* Dublin antibodies using BTM samples. Samples were transported in refrigerated milk tubes with preservatives (ProClin 150, KABE LABORTECHNIK GmbH, Nümbrecht, Germany). Upon arrival at the laboratory, samples were stored at 5 ± 3 °C until a cream layer had formed, then milk serum was pipetted from the bottom of the tubes using steel needles via a pipetting robot (Freedom Evo, TECAN Group Ltd., Männedorf, Switzerland) into deep well plates for storage (VWR International, LLC, Radnor, USA).

### Serological detection of antibodies in BTM samples

Antibody testing of BTM samples was performed at the AGES Institute for Veterinary Disease Control (IVET) Linz. According to the manufacturers’ instructions, 200 µl of BTM samples and 10 µl of undiluted positive and negative controls were used in the ELISA. An automated microplate washer (Biotek 405 LS Washer, Agilent Technologies Inc., Santa Clara, CA, USA) was used to wash the microtiter plate between each incubation step. Photometric analysis was performed by using the Sunrise™ absorbance reader and Magellan™ software (TECAN Group Ltd). The optical density (OD) of the wells was measured at 450 nm (OD_450_) and sample results were calculated as the sample to positive ratio (S/P%). Sample results were interpreted according to the manufacturer’s recommendations, with results of S/P% > 30% considered positive and results of S/P% ≤30% considered negative. The correlation between S/P% values and the antibody titer of a herd was described as semi-quantitative results on a scale with the following divisions: S/P% value of ≤ 30% is indicative of a negative (-) result, representing either the absence or a very weak infection. A value of S/P% > 30% ≤ 80% is indicative of a low-level infection (+), with a prevalence of < 20% affected. A value of S/P% > 80% < 150% is indicative of a moderate infection (++), with a prevalence between 20 and 50% affected. Finally, a value of S/P% ≥ 150% is indicative of a heavy infected herd (+++), with a prevalence of > 50% affected.

In addition, data on *S.* Dublin AB analysis were obtained from Hofer et al. (2024) where AB titers out of BTM samples were determined by ELISA (PrioCHECK *Salmonella* Antibody ELISA Dublin; Thermo Scientific, Walham, USA) as previously described. Briefly, the OD was measured at 450 nm (OD_450_) and sample results were calculated with corrected OD_450_ values of the test sample and positive control, expressed as percent positivity (PP). Percent positivity values ≥ 35% and ≥ 100% were categorized as positive and highly positive, respectively [48].

### Statistical analysis

The data were organized and stored in Microsoft Excel 2010 (Microsoft, Washington, USA) for the purpose of data collection and descriptive analysis. Subsequent analytical statistics were performed using IBM SPSS^®^ Statistics v29 (IBM, New York, USA) and R version 4.3.2 [50]. Results were considered significant at *p* < 0.05.

To evaluate *F. hepatica* antibody responses in Brown Swiss, Simmental and Holstein cattle, analysis of variance (ANOVA) was conducted in the group of alpine pastured cows and in the group of grazing cows without alpine pasture. Correlation between *F. hepatica* (S/P%) and *S.* Dublin (percent positivity, PP) was analyzed using Spearman´s rank correlation.

### Sensitivity analysis by simulation approach on grazing classification

As described above, it was not possible to determine which of the farms registered in the ÖPUL-Weide program actually grazed their dairy cows on lowland pasture. However, since *Fasciola* AB levels for this study were obtained from dairy cows, misclassifying farms as “grazing” when their dairy cows were not or only minimally grazed, would have introduced substantial bias into the risk models. To address this uncertainty, additional data from the national agricultural structure survey were used, which indicated that only 6% of dairy farms in Tyrol do not graze their cows or do so for less than three months annually [47]. This proportion corresponds to approximately 169 dairy farms. Among these, 71 farms were already identified as non-grazing based on the absence of both alpine and ÖPUL-Weide registration. Thus, it was estimated that 98 farms registered under ÖPUL-Weide were likely misclassified as grazing dairy cows. This misclassification was corrected by assuming that 98 of the 896 farms initially classified as “lowland pasture” farms (i.e., ÖPUL-Weide without alpine grazing) were likely not grazing their dairy cows.

Since the specific farms misclassified could not be individually identified, a simulation approach was employed. To address the uncertainty in the classification of non-alpine farms, we performed a frequency-matched stratified resampling of the ambiguous group. Specifically, in each of 200 imputations, 98 non-alpine farms were drawn such that the distribution of *F. hepatica* antibody levels (negative, low, medium, high) matched the distribution observed in confirmed non-grazing farms. Each resampled dataset was then combined with the alpine farms, and risk factor analysis were conducted on all 200 datasets. Risk estimates were subsequently averaged across imputations. The number of imputations (*n* = 200) was chosen a priori as a conservative value to ensure stable point estimates and standard errors from the resampling-based sensitivity analysis; recent methodological work has shown that achieving replicable standard error estimates may require substantially more imputations than the small numbers (e.g. 5–10) traditionally used in applied epidemiological studies [51].

### Model development

#### Risk factors for Fasciola infection

To identify potential risk factors associated with *F. hepatica* seropositivity, a binary logistic regression was conducted using *F. hepatica* serostatus (positive/negative) as the dependent variable, while pasture management data, farm-related variables (number of dairy cows, organic or conventional farm management) and *S.* Dublin seropositivity were included in the model. To investigate the influence of the factors described above on the intensity of antibody response, a univariate linear mixed model was applied using the continuous S/P% value as the dependent variable. Before model fitting, both logical and statistical assessments of collinearity were conducted. In cases where collinearity was identified, the variable considered most biological relevant for explaining the outcome was retained in the original model. Independent variables considered for inclusion in the models included alpine farming-related factors, further farm characteristics such as herd size and breed, as well as *S.* Dublin seropositivity, while the district variable was integrated as random effect. Consequently, the calculated Intraclass Correlation Coefficient (ICC) reported how much of the total variance was explained by differences between districts. In addition, the model was checked visually for normality of residuals and quantitatively for heteroscedasticity (Additional file 2). Subsequently, variables were evaluated using the Bayesian Information Criterion (BIC), and those that reduced model fit were excluded.

#### Association of production parameters with Fasciola infection

A multivariate regression model was built to identify independent variables associated with the dependent variables (milk yield kg, fat% and protein%). The independent variables included S/P%, inter-calving interval, the breed, the number of dairy cows (herd size), the place of milk production during the pasturing season (at farm or at alpine area), alpine pasturing of dairy cows (herd level percentage), and whether producing was organic or not.

Subsequently, multiple-linear regression models were built to analyze the association between *F. hepatica* infection and milk production parameters (milk yield, butter fat content and milk protein content) over a 305-day typical lactation period [52], while each parameter was calculated independently. Explanatory variables remaining in the model were selected based on logical considerations, assessment of collinearity, and the BIC, as described above.

An overall analysis was conducted on the entire dataset without stratification by production system. However, given the considerable heterogeneity among production systems (e.g. challenging conditions of milk production in mountainous regions) and their likely substantial influence on milk yield, models investigating the association between liver fluke antibody titers and production parameters were also conducted separately for each production system to adjust for bias.

The following multiple-linear regression models were built for this purpose: 1. for the production systems separately: grazing of dairy cows on alpine pasture and grazing of dairy cows without alpine pasture (for this the 10 simulated datasets were used as described above); 2, for the following breeds separately: Brown Swiss, Simmental, and Holstein cattle all separately (only for the alpine pasturing system); 3, for different milk yield levels to estimate milk production losses in relation to infection intensity in the group of alpine pastured cows.

By dividing farms into quartiles based on milk yield, the infection effect across different production levels were compared.

The regression coefficient (b) derived from the models was multiplied by (a) the interquartile range of S/P% values (Q1 to Q3 (= 195.6)), and (b) the extended range from Q1 to the maximum of S/P% (= 340.9). Afterwards loss in milk yield was divided by 305 days in order to get the daily loss (in kg) in lactation period. In addition, a univariate linear mixed model was implemented, considering inter-calving interval as dependent variable, which was also checked visually for normality of residuals and quantitatively heteroscedasticity (Additional file 3). Independent variables considered for inclusion in the model included alpine farming-related factors, further farm characteristics such as the number of dairy cows and the breed, as well as *S.* Dublin seropositivity. In addition, the district was integrated as random effect reporting the ICC.

## Results

### Farm characteristics

Descriptive analysis showed that, on average, 16.1 dairy cows were kept per farm. The majority of farms kept Simmental cattle (65.1%), with 98.6% practicing alpine farming, 66.5% keeping dairy cows on alpine pastures, and 29.3% milked cows during alpine pasturing. Communal alpine pastures were used by 85.5% of farms. The average annual milk yield was 7209.7 kg per milking cow (Tables 1, 2 and 3).

**Table 1:** Milk yield and herd size of dairy farms included in this study (*n* = 3148). SD: standard deviation, 95% CI: 95% confidence interval

Variable	Mean ± SD	95% CI	Median	Interquartile range
Milk yield [kg]	7209.7 ± 1514.4	7156.7–7262.6	7170.0	1867.5
Relative milk fat content [%]	4.1 ± 0.4	4.08–4.10	4.1	0.3
Relative milk protein content [%]	3.4 ± 0.2	3.38–3.39	3.4	0.2
Number dairy cows	16.1 ± 13.1	15.7–16.6	12.6	11.4

**Table 2:** Descriptive summary of pasture management on dairy farms included in this study (*n* = 2805)

Variables	Category	Number of dairy farms
Milk production (place)	home	1916
	alpine pasture and home	794
	no data available	95
Dairy cows alpine pasture	yes	1838
	no	967
Communal alpine pasture	yes	1571
	no	267
Alpine pasture general	yes	2766
	no	39
Pasturing of dairy cows	alpine pasturing	1838
	lowland pasturing^a^	896
	no pasturing	71
Organic	yes	677
	no	2034
	no data available	94
Total		2805

^a^ Includes 98 farms with no pasturing, but not individually identifiable

**Table 3:** Distribution of different dairy breeds pasturing on alpine areas; *F. hepatica* positive dairy farms are shown in brackets. *Fasciola hepatica* infections are not shown for breeds occurring on fewer than 100 farms due to data protection

Breed	Number of dairy farms	Number of *F. hepatica* positive farms*	
		Alpine pasturing by dairy cows	Milking on alpine area
Brown Swiss	606	363 (320)	141 (137)
Simmental	1,827	1279 (1232)	591 (568)
Tyrolean Grey	135	84 (69)	9 (7)
Holstein	134	47 (46)	17 (17)
Jersey	19	10	3
Original Braunvieh	29	14	2
Original Pinzgauer	32	21	15
Pinzgauer	18	15	13
Pustertaler Sprintzen	1	1	1
Tuxer	4	4	2

### Prevalence of F. hepatica in Tyrol and S/P% distribution in 2023

**Fig. 1:**
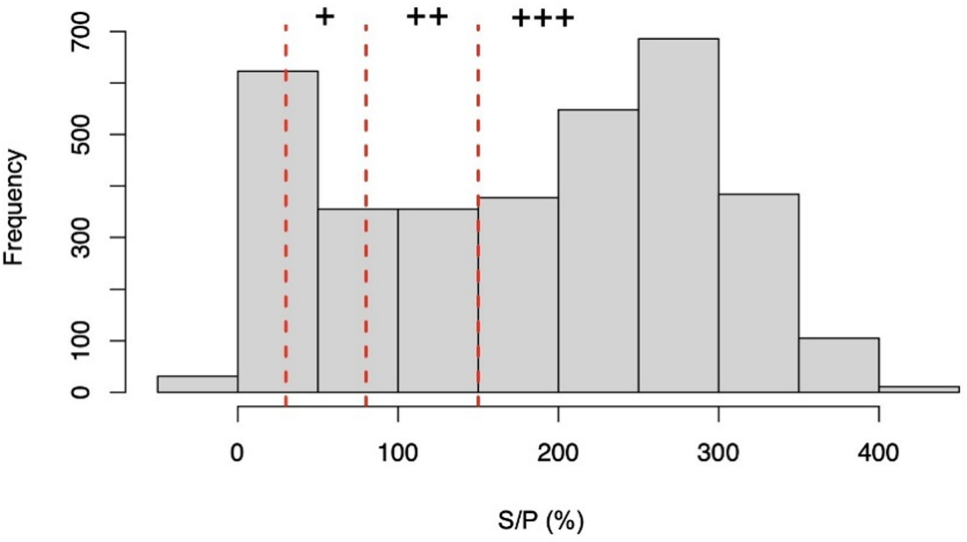
Frequency histogram of bulk tank milk (BTM) samples obtained from the antibody ELISA test for *F. hepatica* (S/P%) from Tyrol (Austria) in 2023 is presented. Dashed lines represent the different category cut-offs e.g., S/P (%) > 30% (low titers, +), S/P (%) ≥ 80% (medium titers, ++), S/P (%) ≥ 150 (high titers, +++)

**Fig. 2:**
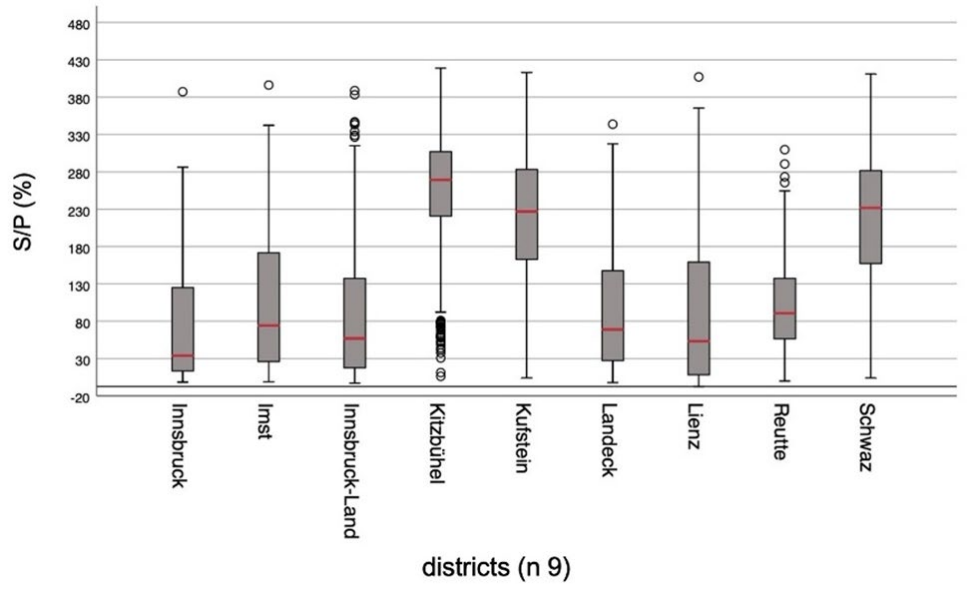
Box and Whisker plots of *Fasciola hepatica* IDEXX S/P% for each district in Tyrol, Austria. The boxes represent the interquartile range (25th – 75th percentile) with the median in red shown as a horizontal line. The whiskers indicate values within 1.5 x interquartile range, while points beyond this range denote outliers

The final dataset contained results of 3645 individual herds. Overall, 86.1% (3140/3645) BTM samples were positive for liver fluke ABs (Table 4). In total, 505 (13.9%) were categorized negative, 424 (11.6%) low, 512 (14.0%) moderate, and 2204 (60.5%) high. An S/P% value of 250–300 had the highest frequency (Fig. 1). The highest titers on average were demonstrated in Kitzbühel, Kufstein and Schwaz (Fig. 2). Antibody titers at district level are shown in Additional file 4.

**Table 4:** Descriptive analysis of bulk tank milk (BTM) test results for F. hepatica in all nine Tyrolian districts

District	*n*	Negative	Positive AB titer			Prevalence (%)
			Low	Medium	High	
Innsbruck	17	7	3	3	4	58.8
Imst	179	47	47	33	52	73.7
Innsbruck-Land	493	168	121	92	112	65.9
Kitzbühel	751	2	23	48	678	99.7
Kufstein	636	16	30	97	493	97.5
Landeck	177	46	54	34	43	74.4
Lienz	408	177	64	60	107	56.6
Reutte	73	11	21	27	14	84.9
Schwaz	911	31	61	118	701	96.6
Total	3645	505	424	512	2204	86.1

When ANOVA was conducted, significant titer differences were observed between the breeds, being highest in alpine pastured Simmental, followed by Holstein and Brown Swiss. This difference was also observed in the group of lowland grazing cows between both Brown Swiss and Holstein, and Brown Swiss and Simmental, but not between Holstein and Simmental. Titers were highest in Simmental and Holstein followed by Brown Swiss (Table 5).

**Table 5:** Average liver fluke antibody titers in pastured animals on alpine and lowland areas. In addition to ANOVA, post-hoc test (Bonferroni) was conducted between each breed. In lowland pastured population randomized farms (*n* 98) were excluded. S.D.: standard deviation

Breed	Alpine pastured		Comparison	*p* Value (Bonferroni)
	*Fasciola hepatica* - antibody titer			
	Mean	S.D.		
Brown Swiss	163.6	99.9	Brown Swiss vs. Simmental	< 0.001*
Simmental	243.8	85.5	Brown Swiss vs. Holstein	0.014*
Holstein	206.1	98.2	Simmental vs. Holstein	0.029*
	**Lowland pastured**			
Brown Swiss	89.3	80.3	Brown Swiss vs. Simmental	< 0.001*
Simmental	147.7	98.3	Brown Swiss vs. Holstein	< 0.001*
Holstein	146.6	94.7	Simmental vs. Holstein	1.0

### Comparison of F. hepatica results in 2005 and 2023

**Fig. 3:**
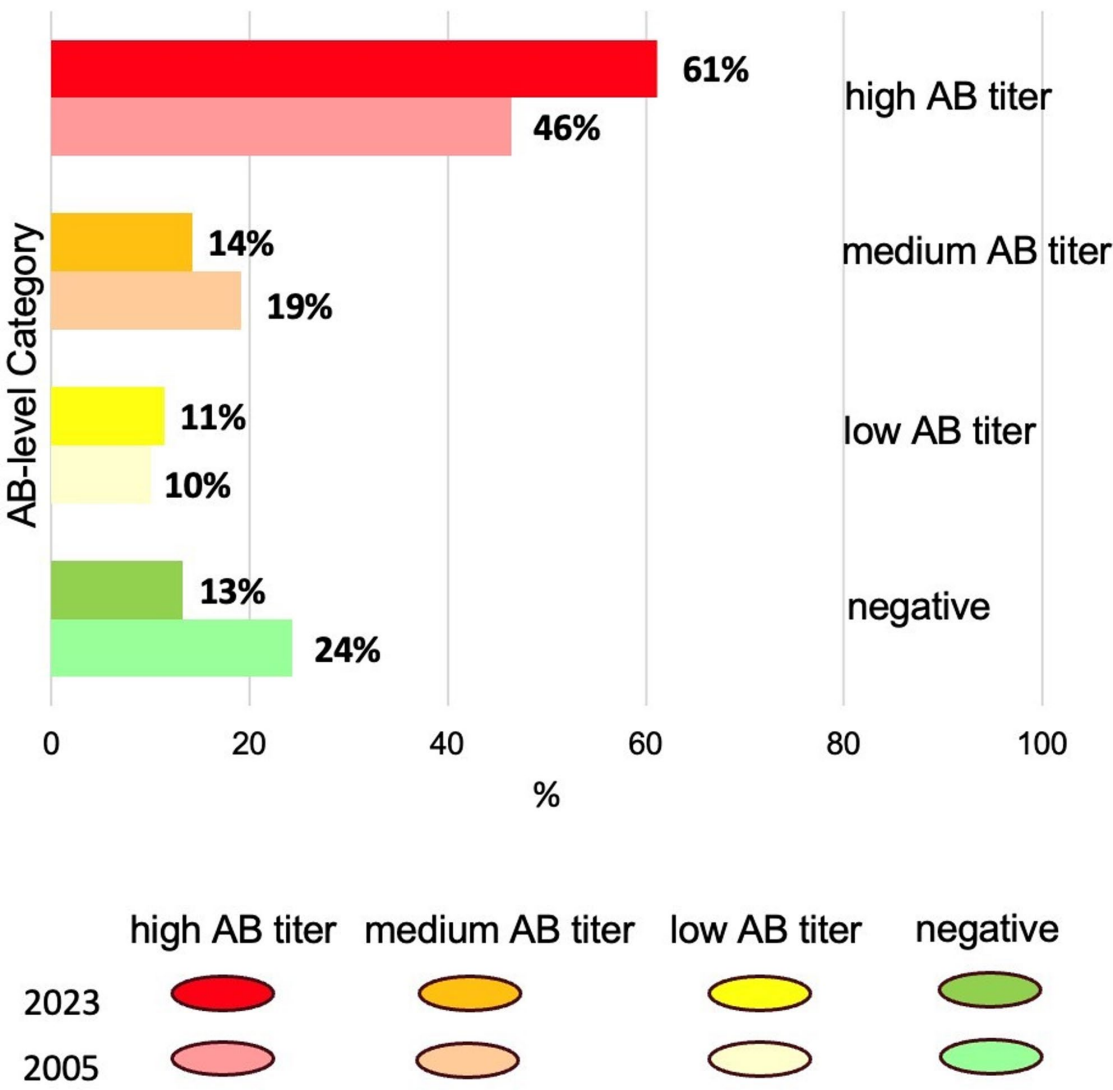
Changes of anti- *F. hepatica* antibody detection on 2855 dairy farms tested in 2005 and 2023

In total 4657 and 3645 bulk tank milk samples were analyzed for liver fluke AB with the same commercial ELISA in 2005 [39] and 2023, respectively. Overall, 2855 individual herds were sampled in both years. Of these 76% (2005) and 87% (2023) BTM samples indicated the presence of *F. hepatica* infections (Additional file 5), while 10% (2005) and 11% (2023) were categorized in the low AB titer category (+); 19% (2005) and 14% (2023) were listed in the moderate category (++); and 46% (2005) and 61% (2023) were listed in high AB titer category (+++) (Fig. 3).

In total, 1638 farms remained in the same AB titer category; 294 (17.9%) negative (-), 75 (4.6%) low (+), 110 (6.7%) moderate (++) and 1159 (71.0%) high category (+++). By contrast, 397 farms seroconverted from negative to positive, while 85 did the opposite. In both years, the category “high antibody titer” dominated and was increased from 2005 to 2023 by 15%, while the category “negative” showed the reverse (Fig. 3). A descriptive analysis demonstrated increasing prevalences in all districts in Tyrol, except in regions where prevalence was already very high, leaving little room for further increase (Additional file 6).

### Comparison of results for S. Dublin AB (2022) and F. hepatica AB (2023)

**Fig. 4:**
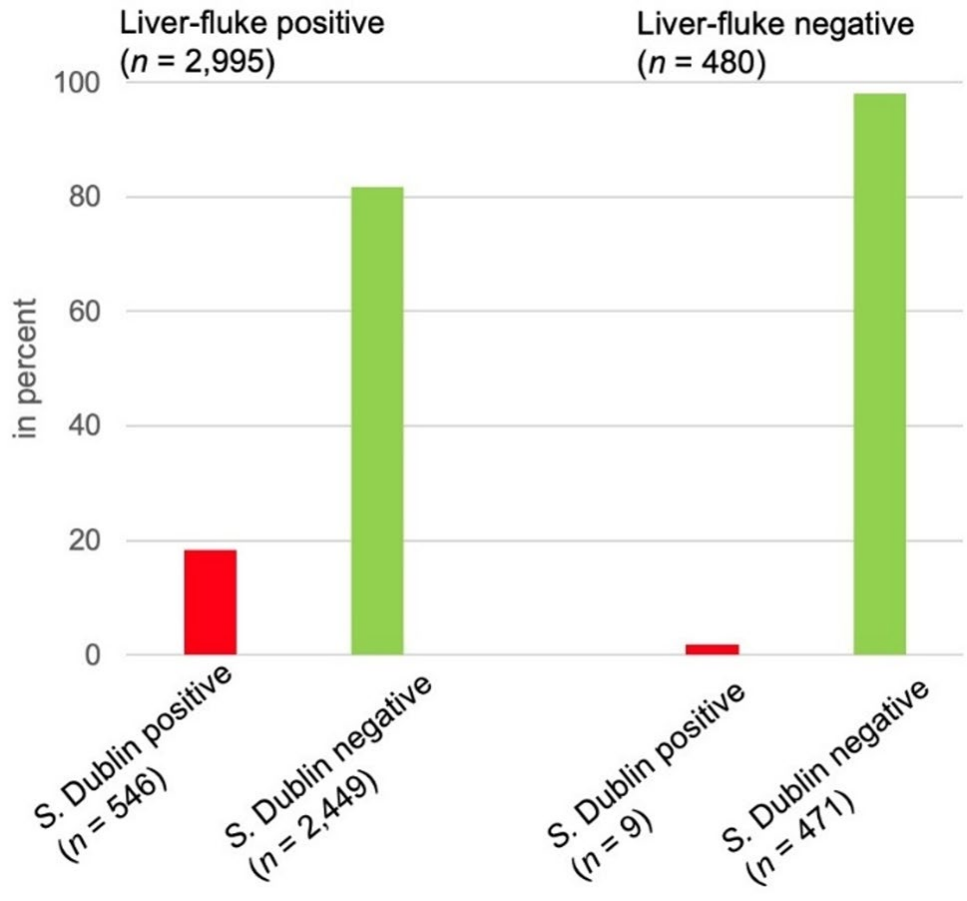
Bulk tank milk (BTM) results for anti-*F. hepatica* and anti-*S.* Dublin antibodies

In 2022, 3670 BTM samples were examined for *S.* Dublin-specific ABs [48]. A total of 3475 dairy farms with BTM results from 2022 (*S.* Dublin AB) and 2023 (*F. hepatica* AB) were compared. Overall, 16.0% of these 3475 farms tested positive for *S.* Dublin specific ABs, while 86.2% samples showed positive results for *F. hepatica* specific ABs. 98% (98.4%) of herds positive for *S.* Dublin showed the presence of liver fluke ABs, while 1.6% positive *S.* Dublin herds were detected in samples from liver fluke negative farms (Fig. 4). Data showed a positive correlation between *F. hepatica* ABs and *S.* Dublin ABs (*r* = 0.406; *p* < 0.001).

### Risk factors associated with F. hepatica infection

#### Binary logistic regression model

94.1%of the farms with dairy cows on alpine pasture tested positive for *F. hepatica*, compared with 73.0% of farms without this practice. This difference was statistically significant in simulated populations with an odds ratio (OR) of 4.7 (Table 6). 96.2% of farms milking on alpine sites were liver fluke positive, while 83.8% of those without milking on mountainous areas were positive resulting in a significant OR of 1.7. In addition, the findings revealed a statistically significant association (OR = 1.7) for *F. hepatica* seropositivity when animals were pastured on communal alpine pastures (i.e., shared grazing areas with livestock from multiple farms). Furthermore, there was an 9.0-fold increased risk (OR = 9.0) to be *F. hepatica* positive when *S.* Dublin was present.

**Table 6:** Binary logistic regression model assessing the association with *F. hepatica* infection status (positive = 1, negative = 0). For this model a frequency-matched stratified resampling was conducted († 98 farms out of 896 were excluded) and repeated for 200 times, in which the average is presented for each variable. Significant associations were observed in five out of seven variables and marked with an asterisk. The number of positive farms is shown in brackets. b: unstandardized coefficient; SE: standard error, n.a.: not analyzed

*Fasciola hepatica* [0/1]	number of farms (*FasciolaFasciola*-positive)	b	SE	*p* Value	Exp (B)
Milk production (place) [0/1]					
[0] home	1916 (1605)				
[1] alpine pasture and home	794 (762)	0.518	0.232	0.026*	1.678
Communal alpine pasture [0/1]					
[0] no	267 (253)				
[1] yes	1571 (1476)	0.517	0.232	0.026*	1.677
Alpine pasture general [0/1]					
[0] no	39 (29)				
[1] yes	2766 (2406)	0.036	0.486	0.942	1.036
Dairy cows on alpine pasture [0/1]					
[0] lowland pasturing †	798 (n.a.)				
[1] alpine pasturing	1838 (1729)	1.549	0.151	< 0.001*	4.709
Number of dairy cows	n.a.	0.045	0.010	< 0.001*	1.046
Organic [0/1]					
[0] no	2034 (1761)				
[1] yes	677 (606)	0.096	0.171	0.574	1.101
*Salmonella* Dublin [0/1]					
[0] no	2374 (2010)				
[1] yes	431 (425)	2.199	0.515	< 0.001*	9.014

Nagelkerkers R^2^: 0.209

#### Univariate linear mixed model

In the univariate linear mixed model, BTM antibody titers were significantly associated with the proportion of dairy cows on alpine pastures (linear), pasturing on communal alpine areas, as well as with the breed, and *S.* Dublin seropositivity (Table 7). No significant association was observed between S/P% and producing milk on alpine sites or under organic farming conditions. The inter-calving interval, number of dairy cows, and practicing alpine pasturing in general (i.e., with at least one age group on alpine pastures) was not significantly associated with *F. hepatica* AB titers in this model. About 30% of the variation in antibody titers was explained at the district level (ICC = 0.306), showing that farms within the same district were more similar to each other than to farms elsewhere. Additional file 7 provides a comprehensive overview of the 95% confidence intervals for each variable analyzed.

**Table 7:** Univariate linear mixed model showing associations between explanatory variables and the dependent variable (*Fasciola hepatica* antibody level S/P%). The district was used as random effect. Variables marked with an asterisk were significant

Variable	df	F	*p* Value
Herd level (%) dairy cows alpine pasture	1	274.629	< 0.001*
Inter-calving interval	1	1.611	0.204
*S.* Dublin ELISA	1	23.121	< 0.001*
Breed	9	7.156	< 0.001*
Communal alpine pasture	1	16.240	< 0.001*
Number of dairy cows	1	0.091	0.763
Alpine pasture general	1	0.022	0.882
Organic	1	1.262	0.261
Milk production (place)	1	3.142	0.076

Pseudo – R^2^: 0.135

### Association of F. hepatica infection and farm management practices with production data

#### Multivariate regression model

The multivariate linear regression model across the whole population demonstrated a significant association of *F. hepatica* positivity with all milk production parameters except relative protein content. In addition, several factors showed significant associations with milk performance, e.g. inter-calving interval, breed and organic farm management. The relative number of dairy cows pasturing on alpine sites and the herd size showed significant associations with most parameters of milk performance, while no associations were detected for the place of milk production (Table 8).

**Table 8:** Results of the multivariate regression analysis estimating the association between the dependent variables (milk kg, fat%, protein%) and independent variables. Associations marked with an asterisk considered to be significant. (S/P%): *F. hepatica* AB level

Variable	Dependent Variable	df	F	*p* Value
S/P%	Milk yield [kg]	1	12.311	< 0.001*
	Fat%	1	26.368	< 0.001*
	Protein%	1	0.318	0.573
Inter calving interval	Milk yield [kg]	1	170.365	< 0.001*
	Fat%	1	36.235	< 0.001*
	Protein%	1	43.614	< 0.001*
Breed	Milk yield [kg]	9	58.902	< 0.001*
	Fat%	9	35.403	< 0.001*
	Protein%	9	68.944	< 0.001*
Number dairy cows	Milk yield [kg]	1	271.416	< 0.001*
	Fat%	1	2.983	0.084
	Protein%	1	135.552	< 0.001*
Milk production (place)	Milk yield [kg]	1	0.112	0.738
	Fat%	1	2.293	0.130
	Protein%	1	1.733	0.188
Herd level (%) dairy cows alpine pasture	Milk yield [kg]	1	8.840	0.003*
	Fat%	1	0.762	0.383
	Protein%	1	5.237	0.022*
Organic	Milk yield [kg]	1	150.867	< 0.001*
	Fat%	1	30.030	< 0.001*
	Protein%	1	103.296	< 0.001*

R^2^: 0.350

#### Multiple linear regression model

In the analysis of dairy cows with alpine pasture management, an increase in the S/P% across the interquartile range of farms with alpine pasture was associated with a non-significant decrease in annual milk production of 0.22 kg per cow per day (*p* < 0.240). However, there were significant negative associations with fat% (*p* < 0.001) and protein% (*p* < 0.003) (Table 9). Furthermore, milk yield in the extended range decreased by 0.38 kg per cow per day (*p* < 0.240), while relative fat and milk protein content decreased by 0.10% (*p* < 0.001) and 0.03% (*p* < 0.003), respectively.

**Table 9:** Results of the multiple linear regression model to determine the association between *F. hepatica* antibody levels (S/P%) in bulk tank milk (BTM) samples and annual average milk yield, relative fat and protein. This table differentiated between pasturing of dairy cows on alpine sites and pasturing on lowland pastures without alpine access. In repeated simulations, 98 farms from this group were excluded, as these were known to have no pasturing but could not be individually identified. The range of simulations is presented in brackets. The number of dairy cows, the breed, the milk production place, and the inter-calving time were included as additional explanatory variables in the model (not shown). Variables marked with an asterisk considered to be significant. b: unstandardized coefficient; SE: standard error; T: test statistic value, 95% CI: 95% confidence interval (b)

Dependent Variable	Constant	Predictor	b	SE	T	*p* Value	95% CI	
Grazing of dairy cows (on alpine pasture; *n* = 1838)							lower 95% CI	upper 95% CI
Milk yield [kg]	8992.467	S/P%	-0.342	0.291	-1.176	0.240	-0.912	0.228
R^2^ = 0.201								
Fat%	3.791	S/P%	-0.0003	0.000	-4.360	< 0.001*	-0.0005	-0.0002
R^2^ = 0.051								
Protein%	3.115	S/P%	-0.0001	0.000	-2.983	0.003*	-0.0004	-0.0002
R^2^ = 0.096								

Grazing of dairy cows (not on alpine pasture; *n* = 798)								
Milk yield [kg]	9018.429–9162.019	S/P%	(-1.184) / (-0.911)	0.245/0.360	(-4.702) / (-3.635)	< 0.001*	-2.313	-0.240
R^2^ = 0.144–0.186								
Fat%	3.730–3.773	S/P%	(-0.0004) / (-0.0003)	0.000	(-6.526) / (-4.999)	< 0.001*	-0.0005	-0.0001
R^2^ = 0.061–0.067								
Protein%	3.103–3.127	S/P%	(-0.0002) / (-0.0001)	0.000	(-5.064) / (-4.500)	< 0.001*	-0.0003	-0.00008
R^2^ = 0.109–0.118								

When investigating associations between liver fluke AB titers and milk production parameters for different breeds grazing on alpine pastures separately, the present study observed a significant negative association between milk yield and S/P% for Brown Swiss (0.78 kg (*p* = 0.039)) and Simmental cattle (0.49 kg per cow per day (*p* = 0.041)). In the extended range a reduction in daily milk yield was observed with 1.35 kg in Brown Swiss and 0.85 kg in Simmental cattle. By contrast, no significant association with milk yield was detected in Holstein cattle (*p* = 0.079) (Table 10).

**Table 10:** Overview of the multiple-linear regression model analyzing *F. hepatica* antibody levels (S/P%) samples and annual average milk yield, relative fat and protein between different breeds: Brown Swiss, Simmental, Holstein cattle. The number of dairy cows, the milk production place, and the inter-calving interval were included as additional explanatory variables in the model (not shown). The number of dairy herds is presented in brackets with corresponding positive farms. Variables marked (*) considered to be significant. b: unstandardized coefficient, SE: standard error, T: test statistic value, 95% CI: 95% confidence interval (b)

Dependent Variable	Constant	Predictor	b	SE	T	*p* value	95% CI	
Grazing of dairy cows: Brown Swiss (on alpine pasture; *n* = 363; 320 liver fluke pos.)							lower 95% CI	upper 95% CI
Milk yield [kg], R^2^ = 0.090	8470.053	S/P%	-1.212	0.584	-2.075	0.039*	-2.360	-0.063
Fat%, R^2^ = 0.053	3.819	S/P%	-0.0003	0.000	-1.736	0.084	-0.001	0.00003
Protein%, R^2^ = 0.146	3.254	S/P%	7.75E-05	0.000	0.305	0.600	-0.00007	0.0002

Grazing of dairy cows: Simmental (on alpine pasture; *n* = 1279; 1232 liver fluke pos.)								
Milk yield [kg], R^2^ = 0.186	9268.459	S/P%	-0.759	0.371	-2.047	0.041*	-1.487	-0.032
Fat%, R^2^ = 0.034	3.695	S/P%	-0.0003	0.000	-3.759	< 0.001*	-0.001	-0.0002
Protein%, R^2^ = 0.057	3.066	S/P%	2.67E-05	0.000	0.525	0.600	-0.00007	0.0001

Grazing of dairy cows: Holstein (on alpine pasture; *n* = 47; 46 liver fluke pos.)								
Milk yield [kg], R^2^ = 0.277	10346.246	S/P%	3.052	1.692	1.803	0.079	-0.374	6.478
Fat%, R^2^ = 0.018	4.262	S/P%	-0.0002	0.000	-0.525	0.603	-0.001	0.001
Protein%, R^2^ = 0.090	3.144	S/P%	-0.0004	0.000	-1.596	0.119	-0.001	0.0001

In grazing cattle without alpine pasturing, simulations based on ten distinct populations (exclusion of *n* = 98 farms), revealed significant negative associations with all milk production parameters (Table 9).

The results for grazing cows on lowland pastures without alpine pasture (simulated models) showed that an increase in the S/P% across the interquartile range was significantly associated with a decrease in daily milk production between 0.58 and 0.76 kg per cow annually (*p* < 0.001). The loss in butterfat ranged between 0.06% and 0.08% (*p* < 0.001) and milk protein content between 0.02% and 0.04% (*p* < 0.001). Furthermore, milk yield in the extended range decreased by 1.02–1.32 kg per cow per day (*p* < 0.001), while butterfat and milk protein content decreased by 0.10% − 0.14% (*p* < 0.002) and 0.03% − 0.07% (*p* < 0.001), respectively.

When dividing farms into lower 25th percentile, 25th to 75th percentile (interquartile range) and upper 25th percentile based on milk yield no significant association between liver fluke infection and production level was observed (Table 11).

**Table 11:** Multiple-linear-regression shows associations between *F. hepatica* ELISA results and dairy farms divided based on milk yield (kg): the lower 25th percentile (low-yielding), the interquartile range (IQR) (medium-yielding) and the upper 25th percentile (high-yielding). Furthermore, analysis was divided in alpine pastured population and not alpine pastured population. The number of dairy cows, the breed, the milk production place, and the inter-calving time were included as additional explanatory variables in the model (not shown). (S/P%): *F. hepatica* AB level, b: unstandardized coefficient, SE: standard error, T: test statistic value, 95% CI: 95% confidence interval (b)

Variable	Percentile	Constant	Predictor	b	SE	T	*p* Value	95% CI	
Grazing of dairy cows on alpine pasture (*n* = 1838)								lower 95% CI	upper 95% CI
Milk yield [kg]	lower 25th	7175.077	S/P%	0.012	0.305	0.040	0.968	-0.588	0.612
	R^2^ = 0.205								
	IQR	7342.739	S/P%	0.033	0.171	0.195	0.846	-0.303	0.370
	R^2^ = 0.045								
	upper 25th	9036.106	S/P%	-0.473	0.348	-1.359	0.175	-1.156	0.211
	R^2^ = 0.090								

Grazing of dairy cows not on alpine pasture (*n* = 896)									
Milk yield [kg]	lower 25th	6481.990	S/P%	-0.264	0.495	-0.533	0.595	-1.239	0.712
	R^2^ = 0.091								
	IQR	7472.781	S/P%	-0.361	0.277	-1.301	0.194	-0.906	0.184
	R^2^ = 0.048								
	upper 25th	9567.800	S/P%	-0.081	0.681	-0.118	0.906	-1.424	1.263
	R^2^ = 0.156								

#### Univariate linear mixed model

When the inter-calving interval was used as the dependent variable, no significant associations were identified with *F. hepatica* seropositivity or pasturing management, including alpine pasturing in general and the location of milk production (on- farm or on alpine area). However, the inter-calving interval was found to be significantly associated with milk yield, relative fat content, relative protein content, cattle breed, organic farm management, and pasturing of dairy cows on alpine areas (Table 12). About 5% of the variation in antibody titers was explained at the district level (ICC = 0.050), demonstrating that the district showed a minor role in the context of inter-calving interval. Additional file 7 shows a detailed overview of the 95% confidence intervals for each variable analyzed.

**Table 12:** Univariate linear mixed model with inter-calving interval as the dependent variable for the whole population. District is integrated as random effect. Variables marked (*) considered to be significant. (S/P%): *F. hepatica* AB levels

Variable	df	F	*p* value
Milk yield [kg]	1	210.160	< 0.001*
Fat%	1	6.092	0.014*
Protein%	1	79.108	< 0.001*
Herd level (%) dairy cows alpine pasture	1	4.725	0.030*
S/P%	1	0.741	0.390
Breed	9	20.338	< 0.001*
Communal alpine pasture	1	0.812	0.368
Number dairy cows	1	0.023	0.879
Alpine pasture general	1	0.257	0.612
Organic	1	22.887	< 0.001*
Milk production (place)	1	3.356	0.185

Pseudo – R^2^: 0.172

## Discussion

The present study investigated the frequency of *F. hepatica* seropositivity on milk-supplying farms in an alpine region, changes of this parameter over time and possible associations with *S.* Dublin seropositivity, milk performance and various potential risk parameters. The broad dataset enabled robust identification of several risk factors related to alpine farming, *S.* Dublin infection, and production parameters.

### Liver fluke prevalence

With a herd-level prevalence of 86.1%, this study demonstrates that Tyrol ranks amongst the most highly prevalent regions for *F. hepatica* infections in Europe. Similar prevalences were observed in the south-western part of Austria (Carinthia), in north-western Spain, England, Ireland and Wales with herd-level prevalences of 60 to 84%, respectively [12, 53–55]. In Germany with an overall lower prevalence, Alpine regions were also identified as highly endemic with 91.3% and 97.5% in Bad Reichenhall and Bad Tölz, respectively [38]. This is consistent with the findings observed in Switzerland, where an overall herd-level prevalence of 41.3% was determined, two distinct clusters with higher AB levels were situated in Alpine regions and a positive correlation was demonstrated between increasing levels of *F. hepatica* AB levels and higher altitude [40]. In line with this, we were, for the first time, able to demonstrate that specifically alpine pasturing of dairy cows correlated significantly with *F. hepatica* infection. This may be partly explained by a widespread presence of suitable snail habitats and favorable ecological conditions for the fluke development in these areas, combined with typical alpine pasturing practices such as prolonged grazing periods and communal pastures [37] with the latter being identified as a risk factor. Another reason for the high prevalence could be slurry application on alpine sites, which is widely practiced on Tyrolian alpine pastures, and was previously described to represent a risk for the contamination of intermediate host habitats and snail infections at high altitudes [37].

However, no detailed information was available on this practice as well as on the duration of pasturing and anthelmintic use - important factors known to influence *Fasciola* (sero)prevalence [44]. Additional in-depth studies on climate conditions and trends, snail occurrence as well as farm management practices are required to substantiate these assumptions.

Since the samples were collected from almost all farms supplying milk in Tyrol, present results accurately represent the liver fluke herd-level prevalence at the time of sampling. The ELISA AB test is generally reported to have very high sensitivity and specificity [56]. The IDEXX Fasciolosis Verification Test, which was also used in the present study, has been evaluated in several studies with varying results depending on sample type, reference method, and sampling season. For individual cattle samples, when liver dissection was used as the reference method, reported sensitivities ranged from 95% in spring to 82% in autumn [57]. When serum samples from animals with a known infection status were used as the reference, sensitivities of 98% and up to 100% were reported [58–61]. Across studies, specificity was consistently high, ranging from 94% to 100%; with lower values observed when compared to liver dissection as the reference method [11, 58–61]. In contrast, relatively few studies have investigated the diagnostic performance of the IDEXX ELISA using bulk tank milk (BTM) samples. Duscher et al. 2011 [54] reported that a minimum within-herd prevalence of approximately 20% positive animals was required to yield a positive BTM result. Takeuchi-Storm et al. 2021 [62] further demonstrated a strong correlation between individual-animal infection status and BTM ELISA S/P% values; however, this relationship was non-linear, with plateau phases observed at three distinct S/P% ranges. It was therefore suggested to use S/P% values only on categorical level. However, this observation was derived from a single study under specific epidemiological conditions with predominantly Holstein breed. It therefore remains uncertain whether the same thresholds would apply to the study population in alpine regions. In the present dataset the distribution of S/P% values showed a continuous pattern with substantial within- and between-group variability and no clear data-driven cut-offs, suggesting that categorization would result in loss of quantitative information. Also, significant and biologically plausible associations were observed when S/P% was analyzed as a continuous variable, and these findings were consistent with results obtained from the binary regression model. Therefore, to preserve quantitative information and avoid arbitrary cut-offs, S/P% was used as a continuous variable to investigate linear relationships.

One limitation of serotesting is that a positive AB detection result may indicate a history of exposure to the parasite rather than a current infection [63]. The period however, between October and November represents the optimal timeframe for sampling, given that the pasture is often most contaminated with metacercariae in late summer, so that, given a seroconversion within 2 to 4 weeks post infection (dependent on the infectious dose of metacercariae), the highest seroprevalence is to be expected in autumn [55, 63–65]. For herd screening, the detection of liver fluke ABs in BTM samples is thus a convenient and non-invasive option and provides information on the extent of infection within the lactating herd [62, 66]. Studies relating infection intensity to antibody levels were conducted in Holstein cows [4, 62] but it is currently unclear whether comparable AB levels at similar infection intensities can be expected in other breeds. Interestingly, Simmental cattle herds had the highest BTM AB titers in our study. It will need further investigations to determine if this reflects higher infection intensities or a stronger humoral immune response in this breed. Breed-related variation in immune responses has been reported previously [67], and resistance to *F. hepatica* infection appears to be heritable to some extent [68]. In addition to genetic factors, differences in the microbiome may also contribute to variation in host response between individual animals [69].

Furthermore, the investigated farms had low average herd size, often resulting in only small numbers of cows contributing to the BTM sample; consequently, results may not be directly comparable with those from many other European countries, where larger herd sizes are common [70], as smaller herds may lead to a disproportionate influence of individual high-antibody cows on BTM results due to reduced dilution effects compared with larger herd [71].

Interestingly, no effect of organic farming on *Fasciola* seroprevalence was observed, consistent with recent findings reported for *Ostertagia ostertag*i in Tyrol [71]. One possible explanation is that management factors frequently assumed to increase parasite prevalence in organic systems (e.g. longer grazing periods or reduced use of fasciolicidal treatments) may not differ markedly between organic and conventional farms in the study region.

### Development of F. hepatica prevalence between 2005 and 2023

The current study, employing the same serological test as the study in 2005, demonstrated an increase in liver fluke infections and AB levels in all districts and the herds with the highest AB levels were situated within the same districts as previously reported by Matt et al. 2007 [39]. This spatial consistency indicates stable environmental conditions favourable for the liver fluke life cycle, such as the persistence of suitable snail habitats, and potentially also unchanged farm management practices within these districts. In comparison with the prevalence at herd-level in 2005, an increase of over 13% was observed. This may be explained by several factors. First, it highlights potential weaknesses in the implementation or effectiveness of current management measures, such as the timing and frequency of anthelminthic treatments, the targeted groups of animals or inadequate pasture management (e.g. failure to avoid high-risk pastures with snail habitats and/or high metacercarial contamination). Improved, regionally adapted control programs might be necessary to address this challenge. Second, environmental factors, particularly climatic conditions, may be favoring the persistence and spread of liver fluke infections. Between the two sampling years of this study, a continuous increase in temperature was recorded in Tyrol, and 2023 was a notably wet year [45]. A connection between climate and liver fluke prevalence, with increased summer rainfall creating favorable conditions for the free-living stages of *F. hepatica* and its intermediate host, has been described [43, 72–74]. However, the relationship between climate and prevalence was not observed in all studies. For instance, Charlier et al. 2013 [75] detected no differences in antibody levels over a three-year sampling period at herd-level, despite notable variations in weather conditions. Additionally, Munita et al. 2016 [44] reported that seasonal weather patterns have just a slight impact on the exposure of *F. hepatica*, whereas a decline in herd-level antibody status was strongly associated with treatment. In Tyrol, however, climate appears to be a major influencing factor for liver fluke prevalence, and this warrants further investigation through the development of a region-specific climate model, as already available for other regions [76–78].

Third, a further cause for the increased prevalence might be the extension of the grazing period. On average, dairy cows spent 5.7 months a year on pasture in 2022 [47], while data from 2010 indicate that cattle spent either on average 4.7 on farm-owned land or 3.6 months on communal pasture [33]. This would be in line with findings observed by Novobilský et al. 2015 [79], who reported that the length of pasturing in heifers was positively correlated with liver fluke antibodies in BTM samples.

### Milk production parameters, breed and pasture management

Over the whole population, we observed a significant association between S/P% and milk yield. However, as this analysis included different housing, grazing conditions and breeds, only the analysis of the stratified data allows a more precise data interpretation. Cows on alpine pastures had the highest *Fasciola* prevalence, while stabled cows with little or no pasture access had the lowest. Considering the difficult conditions of milk production in the mountains (lower-energy diets, greater physical activity of animals, less advanced milking facilites) compared to those under more conventional conditions, differences in milk yield are therefore very likely also explained (and confounded) by housing conditions, rather than only by *Fasciola* infection. Consistently, when the analysis was stratified by housing conditions, no significant negative association with milk yield was detected for cows on alpine pastures, even though a significant decrease was observed on milk quality (relative fat and protein).

However, the deeper stratification by breed revealed that milk yield of dairy cows kept on alpine pastures was indeed negatively associated with *Fasciola* infection. Most likely, differences in antibody response between breeds as described above, masked this effect when all farms were analyzed together.

Against our expectations and unlike Oehm et al. 2023 [41] who observed no *Fasciola*-associated production losses in Simmental cattle, we identified significant negative associations between milk production parameters and *F. hepatica* AB level in alpine pastured Simmental and Brown Swiss cattle, however no significant association was observed in Holstein cattle. One possible explanation for this discrepancy is the large number of animals of those two breeds in our dataset, which provided sufficient statistical power to detect such associations, an effect that might have been missed in studies with fewer herds of those breeds. This hypothesis is further supported by the fact that no significant associations with production losses were found for Holstein cattle in our study, despite previous research reporting a clear impact of *F. hepatica* infection on production parameters in this breed. It is likely that the small number of Holstein herds in our dataset limited our ability to detect such effects. Additionally, it should be considered that 46 out of 47 alpine Holstein farms tested positive for liver fluke, providing insufficient discriminatory power for meaningful comparison. The wide confidence intervals observed for this breed supports this assumption. Additionally, further confounding effects (e.g., anthelminthic treatment) must be considered in interpretation of present results. As there are no regulatory issues or trade implications when animals are infected with *F. hepatica*, the number of farms treating against the liver fluke is unknown.

Interestingly, no production effect was observed in the high-yielding population, irrespective whether cows were grazing on alpine sites or not, which is not in line with previous studies, where high-yielding infected cows were reported to have substantial reduction in milk yield [6, 41]. Considering the seasonal calving management in Tyrolean regions, reduced milk yield is likely due to farm management practices and not (or not only) to liver fluke infection. In the alpine grazing population and in the simulated populations without alpine grazing management, an increase in liver fluke AB titers was significantly associated with a decrease in relative butter fat and relative protein content, which is in agreement with earlier studies [41, 80], but in contrasts other reports where no significant association could be observed [4–6]. However, the detailed stratification by breed, where no associations with protein content were observed, indicated that these association were partly attributable to breed differences rather than to *Fasciola* infections.

In addition to liver fluke antibody titers, the number of dairy cows, the inter-calving interval, the breed, organic farm management, and pasturing of dairy cows on alpine sites as such were significantly associated with milk performance, while the place of milk production did not show significant impact on performance. It should be noted, however, that several variables associated with milk production (such as feeding, anthelminthic treatment, accurate length of the grazing period) were unknown and could thus not be controlled for in our models. Values for S/P% showed no significant association with the inter-calving interval, which is in line with Köstenberger et al. 2017 [80]. In addition, Mezo et al. 2011 [5] and Howell et al. 2015 [6] also did not find associations between fertility parameters and *F. hepatica* exposure, whereas Charlier et al. 2007 [4] showed an increase of 4.7 days in the inter-calving interval when *F. hepatica* ABs were increasing.

To determine the underlying mechanisms of these, sometimes conflicting, observations, interventional studies, e.g. treatment trials, are required to establish a causal relationship. Currently, meaningful comparisons with other studies are challenging due to substantial differences in study design and population, husbandry systems, infection intensities, average milk yield, pasture management, time of sampling, and statistical methodologies. For example, given that 70% of *F. hepatica-*positive farms in Tyrol showed high antibody levels indicating heavier infections, the production losses observed in our study cannot be directly compared to regions with lower BTM antibody titers, since the extent of production loss is closely linked to higher AB titers [4, 6]. This highlights the importance of evaluating production parameters at a regional level to provide more accurate input for models estimating economic impacts for farmers. Additionally, it would be desirable for future studies to follow more harmonized approaches in design and analysis - ideally in the form of harmonized guidelines - to improve comparability across regions and production systems.

### Fasciola hepatica and S. Dublin occurrence

Analysis of the correlation between *F. hepatica* and *S.* Dublin revealed a very strong association, particularly in the binary logistic regression model. In contrast, quantitative measures of infection (AB titers) showed only a weak correlation, suggesting that infection intensity played a relatively minor role in this relationship. While this association has been observed previously [22], this study is the first to confirm it at a large scale by serodiagnosis for both pathogens. Although antibody levels for *S.* Dublin and *Fasciola hepatica* were assessed in different years, substantial changes in herd-level prevalence of *S*. Dublin between consecutive years are unlikely as herd infection status was observed to be relatively stable over time [48], supporting the plausibility of this findings. The observed association between *F. hepatica* and *S.* Dublin could be explained in two ways. One possible explanation is that both *F. hepatica* and *S*. Dublin thrive under similar environmental conditions, leading to their co-occurrence without direct interaction. It is noteworthy that the cluster of *Salmonella* positive herds were detected in the regions where the density of liver fluke positive farms was also high. Mountain pastures were identified as the most likely sites of transmission of *S.* Dublin between cattle of different herd origins [81, 82]. Thus, the habitats of both pathogens, *F. hepatica* and *S.* Dublin, may exhibit similarities, particular in terms of moisture and marshy conditions. Another explanation for this correlation could be a direct biological interaction between these pathogens where *F. hepatica* may play an active role in disseminating *Salmonella* within the host. As the parasite migrates from the intestine to the liver, it could transport the bacteria with it. The tissue damage caused by *F. hepatica* might further facilitate *S.* Dublin colonization in visceral organs.

A limitation of using BTM samples to detect *S*. Dublin antibodies in our study is the potential underestimation of the number of positive herds. This underreporting is likely due to the exclusion of non-lactating animals from the analysis, which may lead to a higher number of undetected cases [83]. Furthermore, the serological test cannot differentiate whether animals within a herd are transiently infected or persistent carriers. Nonetheless, BTM sampling remains a convenient, non-invasive, and cost-effective method of sample collection, making it suitable for large-scale studies, particularly when the observed effect, as in the present study, is strong.

The emergence of *S.* Dublin infections in dairy cattle across Tyrol represent a significant concern for both animal welfare and public health [84]. Infected humans are at increased risk of hospitalization and even death compared to infections with other *Salmonella* serotypes [23]. Therefore, control of *F. hepatica* may also contribute to reducing the prevalence of zoonotic *S.* Dublin infections. The potential connection and interactions between these pathogens warrant further investigation.

## Conclusion

The present study demonstrates that *F. hepatica* remains an increasing challenge for cattle health in Tyrol, suggesting that current control strategies are insufficient. Alpine pasturing was a major risk factor, while breed-specific differences in antibody responses and production losses highlight the importance of management- and breed-adapted interpretation of serological results. Although the drivers of the rising prevalence cannot be fully resolved, climatic changes and intensification of pasturing, which increases the chance of ingesting metacercariae, are likely contributing factors, underlining the need for region-specific epidemiological data. Overall, these findings emphasize the need for integrated control strategies combining herd management, environmental monitoring and predictive modelling to support sustainable alpine dairy farming systems.

## Supplementary Information

Supplementary Material 1.

## Data Availability

The data generated and analyzed during the current study are available from the corresponding author on reasonable request.
