# Supplementary material for: Fasciola hepatica in alpine dairy farming: prevalence trends, risk factors and associations with Salmonella Dublin seropositivity
Source: BMC Vet Res. 2026 Feb 28;22:207. doi: 10.1186/s12917-026-05383-1 (PMC13059266; doi:10.1186/s12917-026-05383-1)
Supplement: Supplementary file 1 — Supplementary Material 1. [file 12917_2026_5383_MOESM1_ESM.pdf]

Additional file 1: The following table provides an overview of the categories included in the study analysis. The number of farms represents those for which this information was available in the dataset.

Category	number of farms
Milk production (place)	2805
Dairy cows alpine pasture (yes/no)	2805
Communal alpine pasture (yes/no)	1838
Alpine pasture general (yes/no)	2805
Number of dairy cows pasturing on alpine area	2805
Organic (yes/no)	2805
ÖPUL-Weide (yes/no)	2805
Number of dairy cows	3148
Milk yield (kg)	3148
Fat%	3148
Protein%	3148
Main breed	3148
Inter-calving interval (in days)	3148
F. hepatica ELISA	3645
S. Dublin ELISA	3475
Tyrolean District	3645

Additional file 2

Assessment of Heteroscedasticity in the Univariate Mixed Model for Fasciola ELISA

Table of Contents

0. Packages

```
library(tidyverse)
library(readxl)
library(janitor)
library(lme4)
library(lmerTest)
library(nlme)
library(patchwork)
library(DHARMa)
```

---

1. Overview

This supplementary report evaluates whether **heteroscedasticity** (non-constant residual variance) is present and statistically significant in the univariate mixed model analyzing *Fasciola hepatica* ELISA values (`fasciola_elisa`). The model (`m`) was fitted assuming **homoscedastic residuals**, and diagnostic analyses were performed to test the validity of this assumption. In particular, we examined the residuals versus fitted values for any systematic variance patterns, quantified the correlation between absolute residuals and fitted values, and performed a formal dispersion test using the DHARMa framework. These complementary approaches were used to determine whether deviations from constant variance were statistically meaningful or negligible.

---

2. Data and model specification

```
dat <- read_xlsx("data/Daten_Gesamt020925_SPSS.xlsx") |>
  clean_names()
dat_for_model <- dat |>
  select(
    fasciola_elisa, milchkuhe_alm_prozent, zwischenkalbezeit, s_befundergeb
nis,
    hauptrasse, gemeinschaftweide_alm, d_kuhzahl, almwirtschaft_generell,
    bio, milchproduktionsort, bezirke
```

```
  ) |>
  mutate(across(
    c(hauptrasse, gemeinschaftweide_alm,
      almwirtschaft_generell, bio,
      milchproduktionsort, bezirke),
    as.factor
  )) |>
  mutate(milchkuhe_alm_prozent = pmin(milchkuhe_alm_prozent, 100)) |>
  drop_na()
```

The variable `milchkuhe_alm_prozent` represents the percentage of milking cows grazing on alpine pastures. Occasionally, values slightly above 100% occur because this variable is derived from an approximate ratio: the number of dairy cows sent to alpine pastures (`Milchkühe_Alm`) divided by the **average herd size** (`d_kuhzahl`) across the year 2023. Since this denominator reflects the annual average rather than the exact number of cows eligible for alpine grazing, some calculated ratios can exceed 100%. To constrain values within a valid range, all entries above 100% were capped at 100%. All rows with missing data in relevant variables were removed to ensure consistent model fitting.

---

3. Homoscedastic model (`m`)

```
m <- lmer(
  fasciola_elisa ~ milchkuhe_alm_prozent + zwischenkalbezeit +
    s_befundergebnis + hauptrasse + gemeinschaftweide_alm +
    d_kuhzahl + almwirtschaft_generell + bio + milchproduktionsort +
    (1 | bezirke),
  data = dat_for_model, REML = TRUE
)

anova(m)

## Type III Analysis of Variance Table with Satterthwaite's method
##                        Sum Sq Mean Sq NumDF  DenDF  F value    Pr(>F)
## milchkuhe_alm_prozent 1706417 1706417     1 2688.4 275.4816 < 2.2e-16 *
**
## zwischenkalbezeit       11653   11653     1 2685.5   1.8813 0.1703001
## s_befundergebnis        74774   74774     1 2686.7  12.0713 0.0005201 *
**
## hauptrasse             426329   47370     9 2685.3   7.6473 3.673e-11 *
**
## gemeinschaftweide_alm  102804  102804     1 2685.2  16.5966 4.757e-05 *
**
## d_kuhzahl                1447    1447     1 2686.6   0.2337 0.6288531
## almwirtschaft_generell    283     283     1 2683.6   0.0457 0.8307333
## bio                     10214   10214     1 2685.9   1.6489 0.1992132
## milchproduktionsort     20771   20771     1 2685.9   3.3533 0.0671831 .
## ---
## Signif. codes:  0 '***' 0.001 '**' 0.01 '*' 0.05 '.' 0.1 ' ' 1
```

---

4. Diagnostic tests for heteroscedasticity

```r
resid_m <- resid(m, type = "pearson")
fitted_m <- fitted(m)

df_resid <- data.frame(
  Model = "m (homoscedastic)",
  Fitted = fitted_m,
  Residual = resid_m
)

ggplot(df_resid, aes(Fitted, Residual)) +
  geom_point(alpha = 0.4, color = "darkgreen") +
  geom_hline(yintercept = 0, color = "red") +
  facet_wrap(~ Model, scales = "free") +
  labs(
    title = "Residuals vs Fitted Values (Model m)",
    x = "Fitted values",
    y = "Pearson residuals"
  ) +
  theme_minimal(base_size = 13)
```

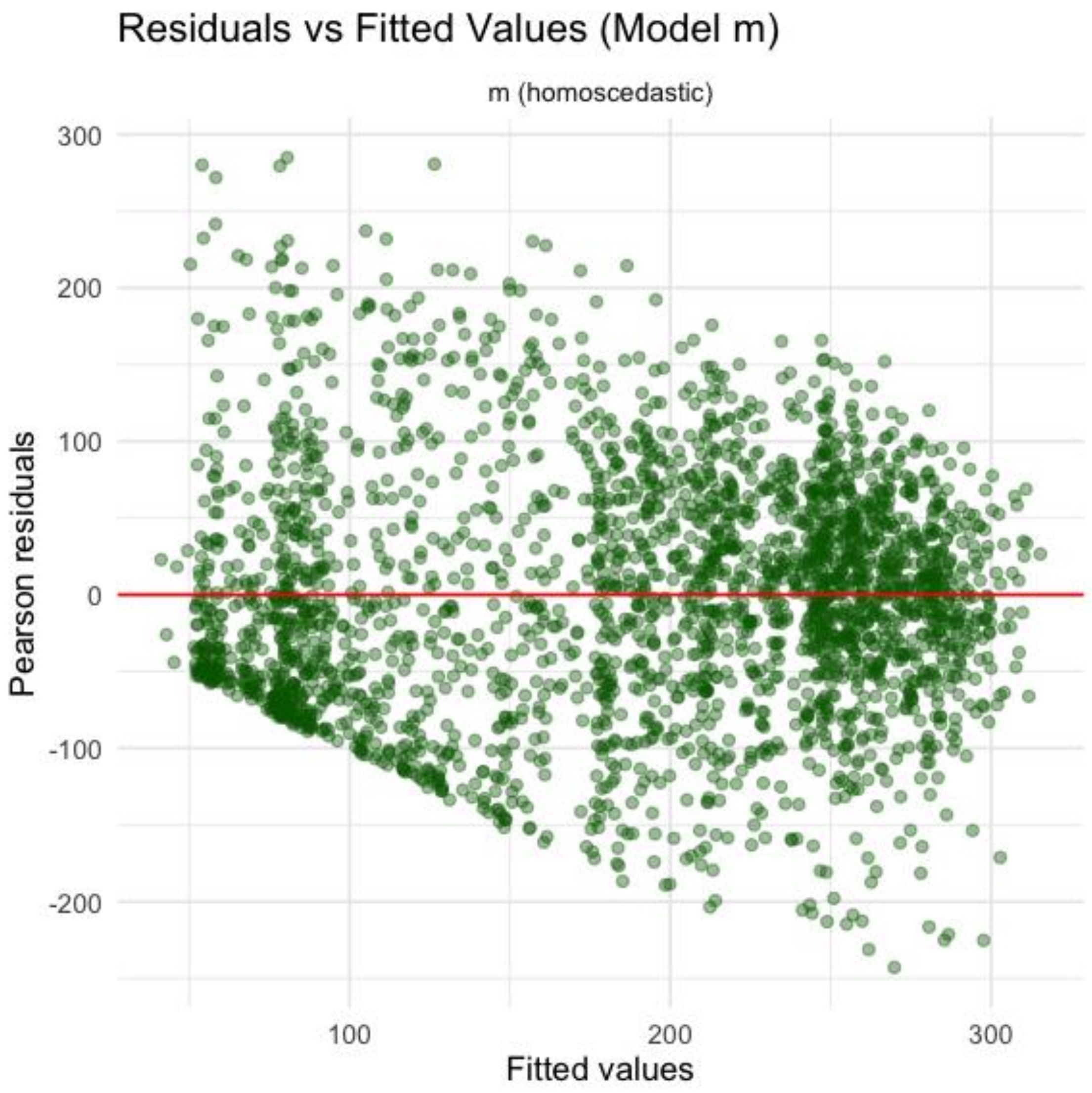

```r
# Correlation between absolute residuals and fitted values
r_corr <- cor(abs(resid(m)), fitted(m))
r_corr
```

```
## [1] -0.1526059
```

```r
# DHARMa dispersion test
simres <- simulateResiduals(m)
testDispersion(simres)
```

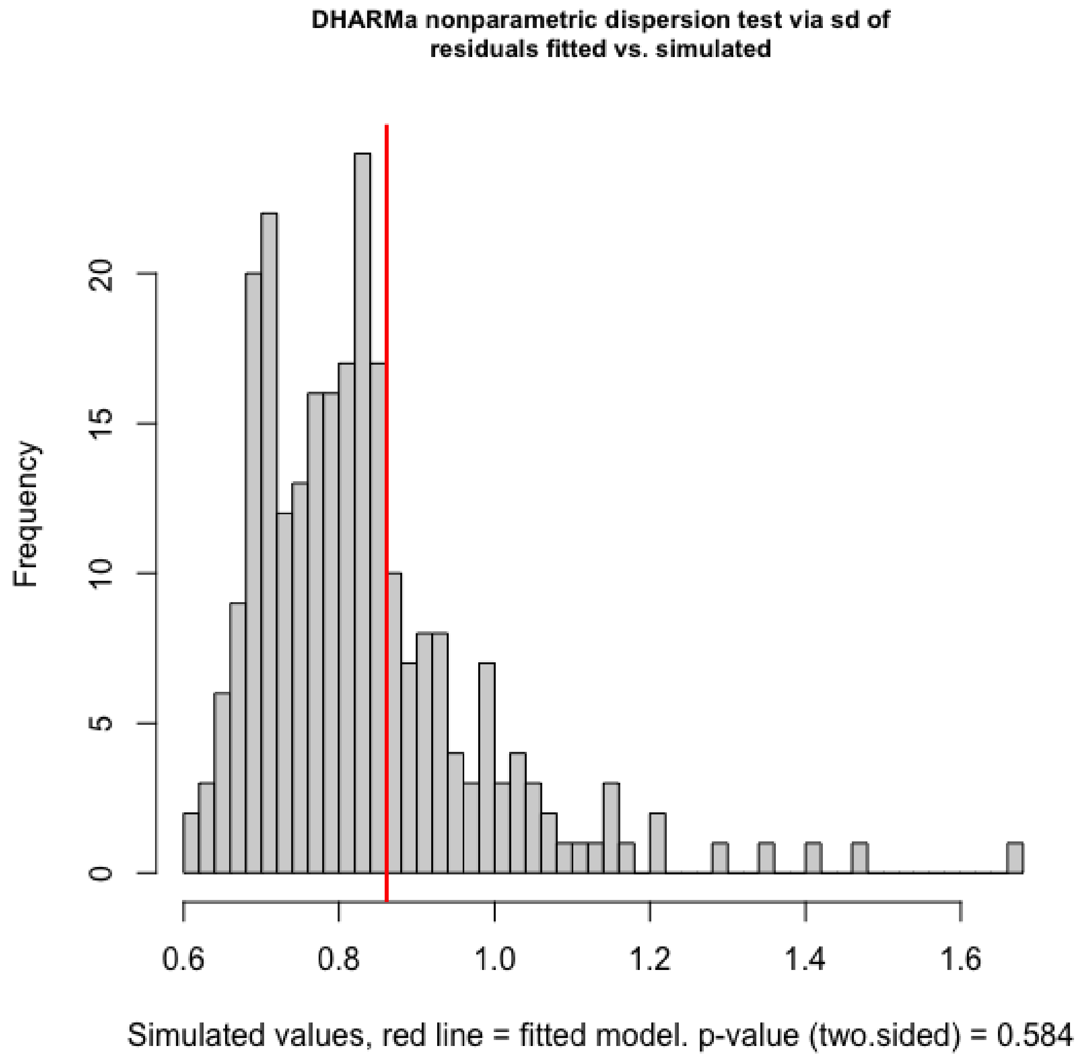


```
##
##  DHARMa nonparametric dispersion test via sd of residuals fitted vs.
##  simulated
##
## data:  simulationOutput
## dispersion = 1.0383, p-value = 0.584
## alternative hypothesis: two.sided
```

---

5. Summary and conclusions

Visual inspection of residuals versus fitted values showed a mild slanted envelope, suggesting a possible change in residual variance with increasing fitted values. However, the vertical spread of residuals remained nearly constant across the fitted range, and no typical funnel-like pattern was observed. The correlation between absolute residuals and fitted values was low ($r = -0.15$), indicating only a weak association between residual magnitude and predicted means. A formal DHARMa dispersion test confirmed that this apparent trend was not statistically significant (dispersion = 1.04, $p = 0.58$). Overall, these diagnostic results indicate that heteroscedasticity in model `m` is minimal and does not meaningfully affect model performance or inference.

✅ *Conclusion:* The assumption of constant residual variance is acceptable, and the homoscedastic model (`m`) is retained for presentation and interpretation in the main manuscript.

Additional file 3

Assessment of Heteroscedasticity in the Univariate Mixed Model for Inter-Calving Interval

Table of Contents

0. Packages

```
library(tidyverse)
library(readxl)
library(janitor)
library(lme4)
library(lmerTest)
library(nlme)
library(patchwork)
library(DHARMa)
```

---

1. Overview

This supplementary report evaluates **heteroscedasticity** in the univariate mixed model analyzing the **inter-calving interval** (`zwischenkalbezeit`). The homoscedastic model (`m2`) assumes constant residual variance, while the heteroscedastic model (`m2_varpower`) allows variance to depend on fitted values using a `varPower` structure.

---

2. Data and model specification

```
dat <- read_xlsx("data/Daten_Gesamt020925_SPSS.xlsx") |>
  clean_names()

dat_for_model2 <- dat |>
  select(
    zwischenkalbezeit, milch_kg, fett_pr, eiweiss_pr,
    milchkuhe_alm_prozent, fasciola_elisa, hauptrasse,
    gemeinschaftweide_alm, d_kuhzahl, almwirtschaft_generell,
    bio, milchproduktionsort, bezirke
  ) |>
  mutate(across(
    c(hauptrasse, gemeinschaftweide_alm,
      almwirtschaft_generell, bio,
      milchproduktionsort, bezirke),
```

```r
    as.factor
  )) |>
  mutate(milchkuhe_alm_prozent = pmin(milchkuhe_alm_prozent, 100)) |>
  drop_na()

cat("Number of observations used in the model: ", nrow(dat_for_model2), "\n
")

## Number of observations used in the model:  2709
```

The variable `milchkuhe_alm_prozent` represents the percentage of milking cows grazing on alpine pastures. Occasionally, values slightly above 100% occur because this variable is derived from an approximate ratio: the number of dairy cows sent to alpine pastures (`Milchkühe_Alm`) divided by the **average herd size** (`d_kuhzahl`) across the year 2023. Since this denominator reflects the annual average rather than the exact number of cows eligible for alpine grazing, some calculated ratios can exceed 100%. To constrain values within a valid range, all entries above 100% were capped at 100%. All rows with missing data in relevant variables were removed to ensure consistent model fitting.

---

3. Homoscedastic model (`m2`)

```r
m2 <- lmer(
  zwischenkalbezeit ~ milch_kg + fett_pr + eiweiss_pr +
    milchkuhe_alm_prozent + fasciola_elisa + hauptrasse +
    gemeinschaftweide_alm + d_kuhzahl + almwirtschaft_generell +
    bio + milchproduktionsort + (1 | bezirke),
  data = dat_for_model2, REML = TRUE
)

anova(m2)

## Type III Analysis of Variance Table with Satterthwaite's method
##                        Sum Sq Mean Sq NumDF  DenDF  F value    Pr(>F)
## milch_kg               382991  382991     1 2684.2 210.1583 < 2.2e-16 **
*
## fett_pr                 11102   11102     1 2686.3   6.0919   0.01364 *
## eiweiss_pr             144163  144163     1 2685.9  79.1066 < 2.2e-16 **
*
## milchkuhe_alm_prozent    8611    8611     1 2677.7   4.7251   0.02981 *
## fasciola_elisa           1351    1351     1 2278.9   0.7411   0.38940
## hauptrasse             332504   36945     9 2635.7  20.2727 < 2.2e-16 **
*
## gemeinschaftweide_alm    1479    1479     1 2688.9   0.8117   0.36771
## d_kuhzahl                  42      42     1 2688.9   0.0232   0.87905
## almwirtschaft_generell    469     469     1 2682.2   0.2573   0.61200
## bio                     42568   42568     1 2686.9  23.3585  1.42e-06 **
*
## milchproduktionsort      3206    3206     1 2687.1   1.7591   0.18485
## ---
## Signif. codes:  0 '***' 0.001 '**' 0.01 '*' 0.05 '.' 0.1 ' ' 1
```

4. Diagnostic tests for heteroscedasticity

```r
resid_m <- resid(m2, type = "pearson")
fitted_m <- fitted(m2)

df_resid <- data.frame(
  Model = "m2 (homoscedastic)",
  Fitted = fitted_m,
  Residual = resid_m
)

ggplot(df_resid, aes(Fitted, Residual)) +
  geom_point(alpha = 0.4, color = "darkgreen") +
  geom_hline(yintercept = 0, color = "red") +
  labs(
    title = "Residuals vs Fitted Values (Model m2)",
    x = "Fitted values",
    y = "Pearson residuals"
  ) +
  theme_minimal(base_size = 13)
```

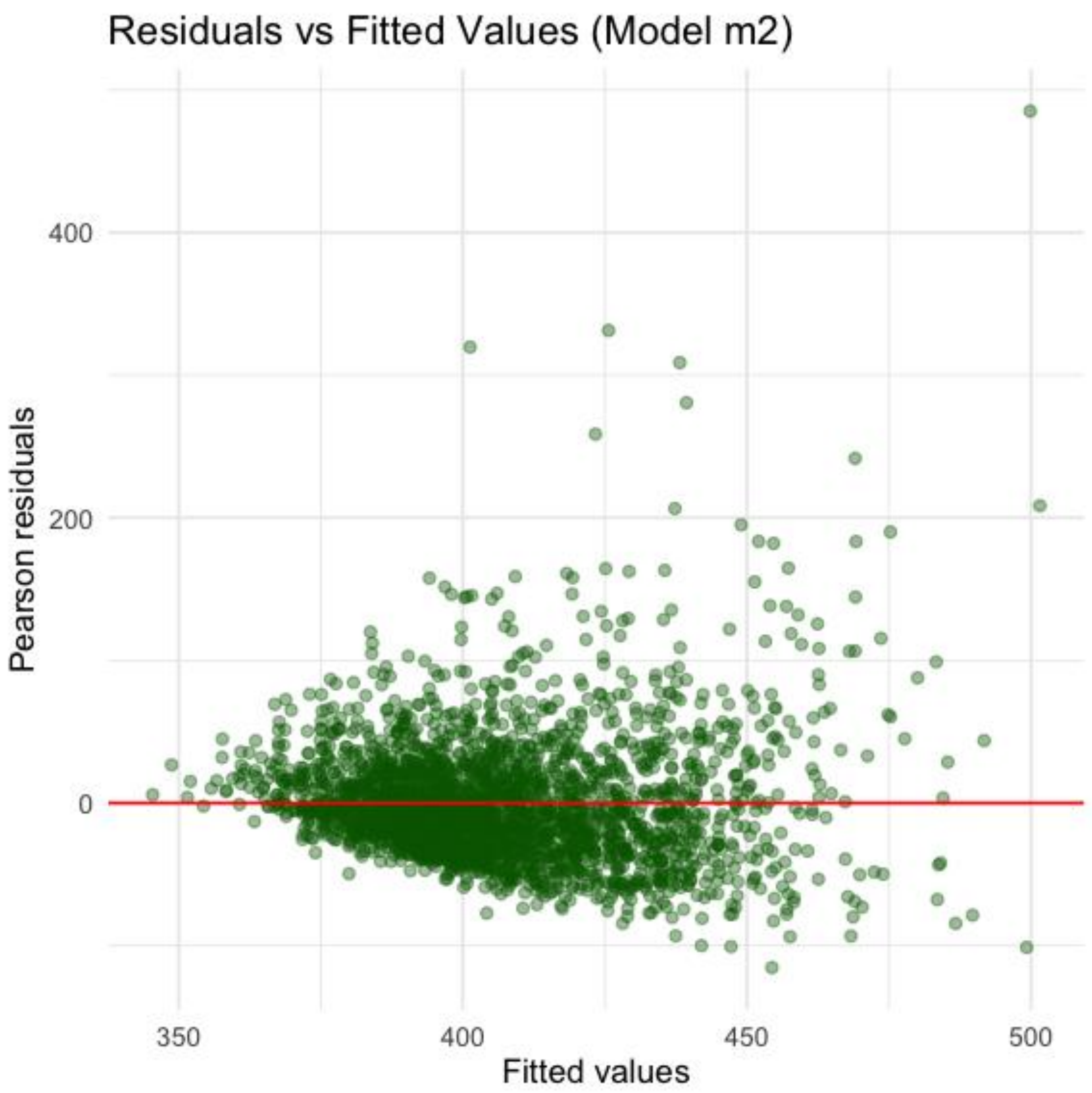


```r
# Correlation between absolute residuals and fitted values
r_corr <- cor(abs(resid(m2)), fitted(m2))
r_corr

## [1] 0.3502275

# DHARMa dispersion test
simres <- simulateResiduals(m2)
testDispersion(simres)
```

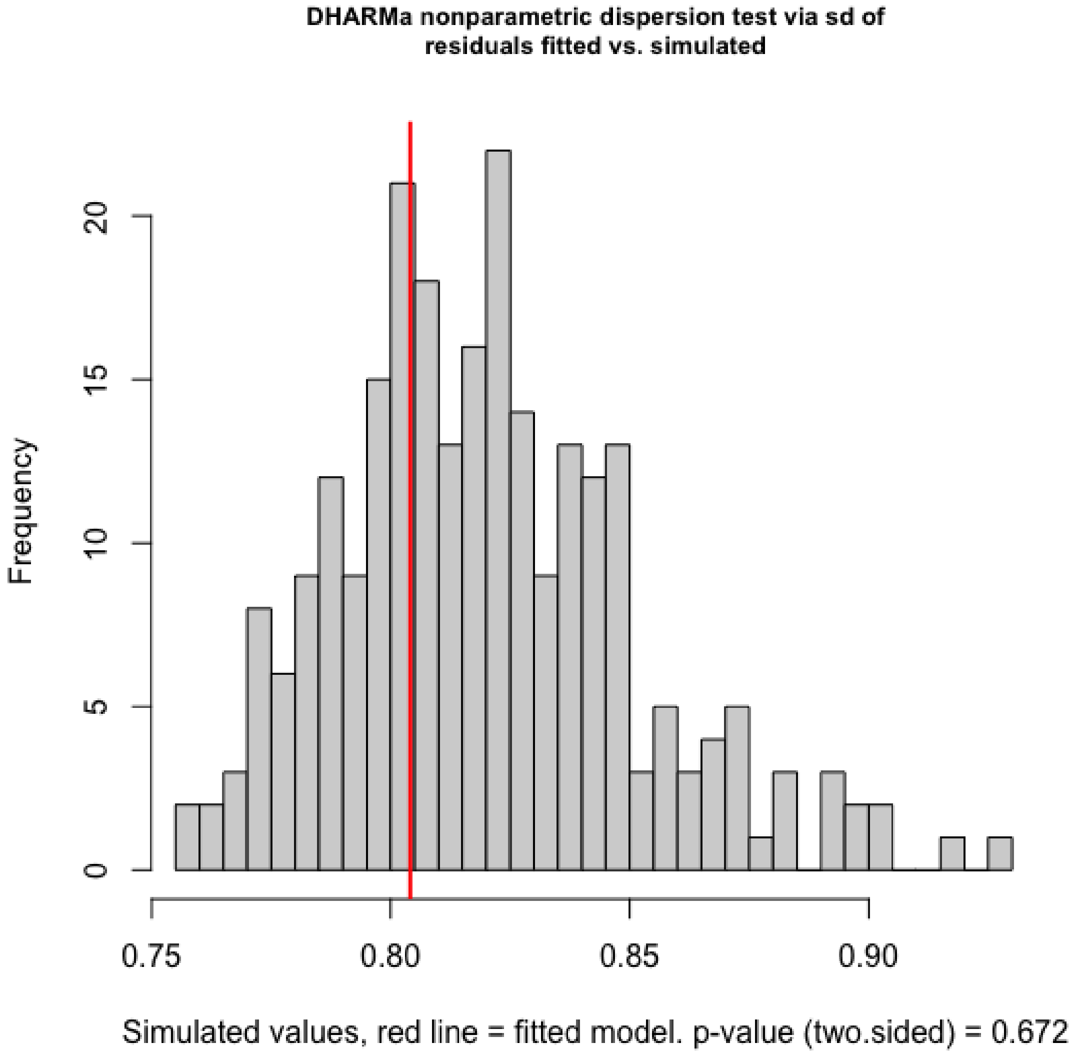


```
##
##  DHARMa nonparametric dispersion test via sd of residuals fitted vs.
##  simulated
##
## data:  simulationOutput
## dispersion = 0.98106, p-value = 0.672
## alternative hypothesis: two.sided
```

Although the DHARMa dispersion test did not indicate significant heteroscedasticity (dispersion = 0.98, p = 0.67), the residuals vs fitted plot displayed a slight funnel-like widening and the correlation between absolute residuals and fitted values was moderate (r = 0.35). These observations suggest a possible increase in residual variance with fitted values, even if not statistically strong. To account for this visual pattern and assess the robustness of parameter estimates, we therefore fitted an alternative model (`m2_varpower`) incorporating a variance-power (`varPower`) structure. This allowed residual variance to increase as a function of the mean while keeping the overall model specification identical.

4. Heteroscedastic model (m2_varpower)

```
m2_varpower <- nlme::lme(
  fixed = zwischenkalbezeit ~ milch_kg + fett_pr + eiweiss_pr +
            milchkuhe_alm_prozent + fasciola_elisa + hauptrasse +
            gemeinschaftweide_alm + d_kuhzahl + almwirtschaft_generell +
            bio + milchproduktionsort,
  random = ~ 1 | bezirke,
  data = dat_for_model2,
  method = "REML",
  weights = varPower(form = ~ fitted(.))
)

anova(m2_varpower, type = "marginal")

##                         numDF denDF   F-value p-value
## (Intercept)                 1  2681 243.23853  <.0001
## milch_kg                    1  2681 145.02089  <.0001
## fett_pr                     1  2681   5.35747  0.0207
## eiweiss_pr                  1  2681  49.74891  <.0001
## milchkuhe_alm_prozent       1  2681   7.88347  0.0050
## fasciola_elisa              1  2681   0.80663  0.3692
## hauptrasse                  9  2681  16.63573  <.0001
## gemeinschaftweide_alm       1  2681   1.15392  0.2828
## d_kuhzahl                   1  2681   0.78395  0.3760
## almwirtschaft_generell      1  2681   0.55653  0.4557
## bio                         1  2681  22.30790  <.0001
## milchproduktionsort         1  2681   2.24529  0.1341
```

The `type = "marginal"` option is required for `nlme::lme` models because it computes F-tests for fixed effects **after accounting for the variance structure**, ensuring that significance reflects the marginal contribution of each predictor under heteroscedasticity.

---

5. Model comparison

```
AIC_table <- data.frame(
  Model = c("m2 (homoscedastic)", "m2_varpower (heteroscedastic)"),
  df = c(length(fixef(m2)) + 1, length(fixef(m2_varpower)) + 1),
  AIC = c(AIC(m2), AIC(m2_varpower)),
  logLik = c(logLik(m2), logLik(m2_varpower))
)
knitr::kable(AIC_table, caption = "Table S1. Model comparison based on AIC
and log-likelihood.")
```

Table S1. Model comparison based on AIC and log-likelihood.

Model	df	AIC	logLik
m2 (homoscedastic)	21	28016.63	-13986.31
m2_varpower (heteroscedastic)	21	27394.68	-13674.34

```r
LR <- 2 * (as.numeric(logLik(m2_varpower)) - as.numeric(logLik(m2)))
pval <- pchisq(LR, df = 1, lower.tail = FALSE)
data.frame(LR_statistic = LR, df = 1, p_value = pval)
```

```
##   LR_statistic df       p_value
## 1     623.9464  1 1.036195e-137
```

The heteroscedastic model (`m2_varpower`) provided a markedly superior fit compared to the homoscedastic model (`m2`), as indicated by a substantially lower AIC (27,405 vs. 28,027) and a significantly higher log-likelihood (LR = 623.94, df = 1, $p < 1 \times 10^{-136}$).

---

6. Diagnostic plots

```r
resid_m2 <- resid(m2, type = "pearson")
fitted_m2 <- fitted(m2)

resid_m2_var <- resid(m2_varpower, type = "pearson", level = 1)
fitted_m2_var <- fitted(m2_varpower, level = 1)

df_resid <- data.frame(
  Model = rep(c("m2 (homoscedastic)", "m2_varpower (heteroscedastic)"),
              times = c(length(resid_m2), length(resid_m2_var))),
  Fitted = c(fitted_m2, fitted_m2_var),
  Residual = c(resid_m2, resid_m2_var)
)

ggplot(df_resid, aes(Fitted, Residual)) +
  geom_point(alpha = 0.4, color = "darkgreen") +
  geom_hline(yintercept = 0, color = "red") +
  facet_wrap(~ Model, scales = "free") +
  labs(title = "Residuals vs Fitted for both models",
       x = "Fitted values", y = "Pearson residuals") +
  theme_minimal(base_size = 13)
```

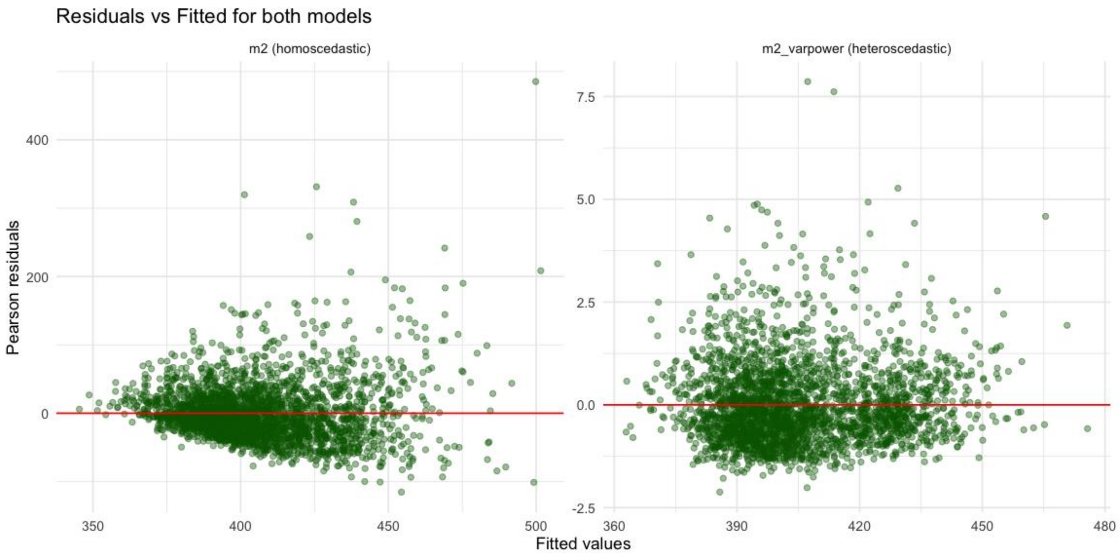

Figure S1. Residuals vs fitted values for the homoscedastic (`m2`) and heteroscedastic (`m2_varpower`) models.

The heteroscedastic model successfully removed the funnel-shaped pattern seen in the homoscedastic model, indicating that variance increases with fitted values were effectively modeled.

---

7. Fixed effects comparison

```
anova_m2 <- anova(m2)
anova_m2_varpower <- anova(m2_varpower, type = "marginal")

tab1 <- data.frame(Predictor = rownames(anova(m2)), anova(m2), row.names =
NULL)
tab2 <- data.frame(Predictor = rownames(anova(m2_varpower, type = "marginal
")),
                   anova(m2_varpower, type = "marginal"), row.names = NULL)
tab2 <- tab2 |> filter(Predictor != "(Intercept)")

comparison <- full_join(tab1, tab2, by = "Predictor", suffix = c("_m2", "_v
arpower"))

final_table <- comparison %>%
  transmute(
    Predictor,
    F_m2 = round(F.value_m2, 2),
    p_m2 = formatC(Pr..F., format = "e", digits = 2),
    F_varpower = round(F.value_varpower, 2),
    p_varpower = formatC(p.value, format = "e", digits = 2)
  )

knitr::kable(final_table,
  caption = "Table S2. Comparison of fixed effects between homoscedastic (m
2) and heteroscedastic (m2_varpower) models."
)
```

Table S2. Comparison of fixed effects between homoscedastic (m2) and heteroscedastic (m2_varpower) models.

Predictor	F_m2	p_m2	F_varpower	p_varpower
milch_kg	210.16	6.58e-46	145.02	0.00e+00
fett_pr	6.09	1.36e-02	5.36	2.07e-02
eiweiss_pr	79.11	1.06e-18	49.75	2.21e-12
milchkuhe_alm_prozent	4.73	2.98e-02	7.88	5.02e-03
fasciola_elisa	0.74	3.89e-01	0.81	3.69e-01
hauptrasse	20.27	2.54e-33	16.64	0.00e+00
gemeinschaftweide_alm	0.81	3.68e-01	1.15	2.83e-01
d_kuhzahl	0.02	8.79e-01	0.78	3.76e-01

Predictor	F_m2	p_m2	F_varpower	p_varpower
almwirtschaft_generell	0.26	6.12e-01	0.56	4.56e-01
bio	23.36	1.42e-06	22.31	2.44e-06
milchproduktionsort	1.76	1.85e-01	2.25	1.34e-01

The same fixed effects remained significant across both models—particularly `milch_kg`, `fett_pr`, `eiweiss_pr`, `milchkuhe_alm_prozent`, `hauptrasse`, and `bio`. This indicates that while heteroscedasticity correction substantially improved fit, it did not alter inference on the main predictors.

---

8. Summary and conclusions

Modeling heteroscedasticity in the inter-calving interval model using a `varPower` variance structure markedly improved model fit and corrected non-constant variance patterns in the residuals. The strong improvement in AIC and log-likelihood (ΔAIC ≈ 620, LR $p < 1 \times 10^{-136}$) confirmed that residual variance increased with fitted values and was effectively captured by the heteroscedastic model.

Despite this adjustment, the **direction and significance of key predictors remained unchanged**: - Milk yield (`milch_kg`), milk composition (`fett_pr`, `eiweiss_pr`), and the proportion of alpine-grazing cows (`milchkuhe_alm_prozent`) retained their strong effects on the inter-calving interval.
- The factor `hauptrasse` (breed) and organic status (`bio`) also remained significant in both models.

✅ *Conclusion:* The heteroscedastic structure improved residual homogeneity and model fit but did not alter the interpretation of fixed effects. Therefore, for consistency with the main analysis, the homoscedastic model (`m2`) is retained for presentation and interpretation in the main text, with heteroscedastic results reported here for completeness.

Additional file 4: Summary statistics for antibody levels against *F. hepatica* (S/P%) in each district of Tyrol collected from dairy herds in October and November 2023.

Variable<br>S/P%	n	Minimum	Percentile<br>Lower 25th	 Median	 Upper 25th	Maximum
Innsbruck	17	-1.6	11.3	33.9	165.6	387.3
Imst	179	-1.1	24.1	74.2	171.2	396.2
Innsbruck-Land	493	-3.0	17.6	57.0	137.0	388.8
Kitzbühel	751	6.3	220.2	269.3	307.0	418.9
Kufstein	636	4.1	162.6	226.9	283.6	413.0
Landeck	177	-2.2	27.1	69.1	147.7	343.8
Lienz	408	-7,5	8	53.3	159.7	407.1
Reutte	73	0.0	55.3	90.7	141.6	309.7
Schwaz	911	4.0	157.2	231.8	281.7	411.0
Overall	3645	-7.0	78.0	199.3	273.6	418.9

Additional file 5: Distribution of four *F. hepatica* antibody level based on antibody detection in 2005 and 2023 from all dairy herds in Tyrol, Austria.

Antibody Category		2005	2023	Total
No to very weak infection	no. of samples	691	379	1070
	% of row	64.6	35.4	100
Low	no. of samples	289	325	614
	% of row	47.1	52.9	100
Medium	no. Samples	545	400	945
	% of row	57.7	42.3	100
Strong	no. Samples	1330	1751	3081
	% of row	43.2	56.8	100
Total	no. Samples	2,855	2,855	5710
	% of row	50	50	100

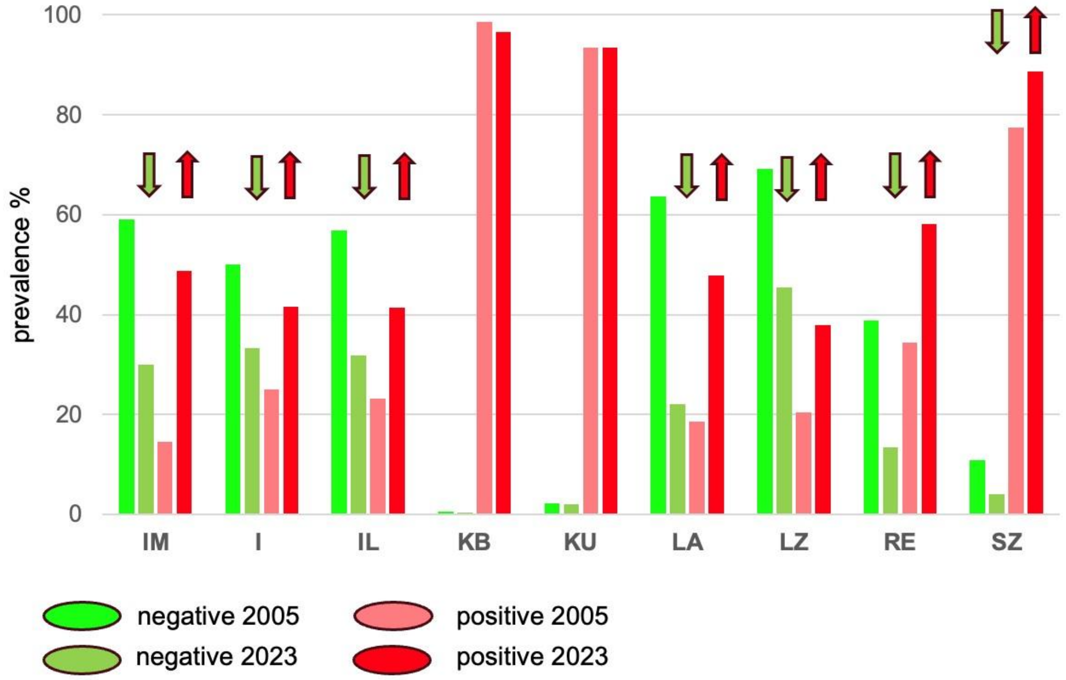

Additional file 6: Descriptive analysis of bulk tank milk (BTM) samples in 2005 and 2023 divided into all nine districts of Tyrol (n = 2855).

(IM: Imst, I: Innsbruck, IL: Innsbruck-Land, KB: Kitzbühel, KU: Kufstein. LA: Landeck, LZ: Lienz, RE: Reutte, SZ: Schwaz; medium and strong infections summarized in positive ones).

Additional file 7: This is an overview of the univariate linear mixed models (Table 7 and Table 12) including estimates and 95% confidence intervals (CI). Marked (*) p values considered significant. n.a.: not analyzed

Dependent Variable: S/P%

**fixed effects**			**95% CI**	
**Breed**	**Estimate**	**p Value**	**lower 95% CI**	**upper 95% CI**
Brown Swiss	-57.189	0.148	-134.769	20.390
Simmental	-23.209	0.556	-100.524	54.106
Tyrolean Grey	-32.168	0.423	-110.811	46.475
Holstein	-26.610	0.508	-105.369	52.149
Jersey	-4.047	0.926	-89.227	81.132
Original Braunvieh	-16.510	0.696	-99.245	66.226
Original Pinzgauer	-32.298	0.441	-114.442	49.845
Pinzgauer	-28.854	0.508	-114.622	56.714
Pustertaler Sprintzen	-12.568	0.886	-184.982	159.845
Tuxer	n.a	n.a	n.a	n.a.
Communal alpine pasture				
no	-19.448	<0.001*	-28.912	-9.985
yes	n.a			
Alpine pasture general				
no	1.953	0.882	-23.941	27.846
yes	n.a			
Organic				
no	-4.199	0.261	-11.531	3.132
yes	n.a			
Milk production place				
home	7.710	0.076	-0.818	16.239
Alpine pasture	n.a			
Herd level (%) dairy cows alpine pasture	0.775	<0.001*	0.683	0.866
Inter-calving interval	0.043	0.204	-0.023	0.109
S. Dublin ELISA	0.197	<0.001*	0.117	0.277
Number of dairy cows	0.042	0.763	-0.230	0.313

Pseudo-R^2: 0.135

Dependent Variable: Inter-calving interval

**fixed effects**			**95% CI**	
	**Estimate**	**p Value**	**lower 95% CI**	**upper 95% CI**
Breed				
Brown Swiss	45.588	0.035*	3.247	87.929
Simmental	28.419	0.187	-13.778	70.616
Tyrolean Grey	2.040	0.925	-40.718	44.798
Holstein	65.349	0.003*	22.161	108.538
Jersey	40.928	0.087	-6.010	87.867
Original Braunvieh	6.187	0.788	-38.818	51.191

Original Pinzgauer	37.052	0.104	-7.610	81.714
Pinzgauer	38.209	0.108	-8.455	54.873
Pustertaler Sprintzen	78.261	0.102	-15.417	171.940
Tuxer	n.a	n.a	n.a	n.a.
Communal alpine pasture				
no	2.368	0.368	-2.786	7.523
yes	n.a			
Alpine pasture general				
no	-3.649	0.612	-17.755	10.457
yes	n.a			
Organic				
no	10.036	<0.001*	5.965	14.108
yes	n.a			
Milk production place				
home	3.129	0.185	-1.496	7.754
Alpine pasture	n.a			
Herd level (%) dairy cows alpine pasture	-0.058	0.030*	-0.110	-0.006
Milk yield [kg]	-0.010	<0.001*	-0.012	-0.009
Fat%	8.037	0.014*	1.652	14.422
Protein%	60.660	<0.001*	-0.230	74.033
S/P%	0.009	0.390	-0.011	0.029
Number of dairy cows	0.012	0.879	-0.143	0.167

Pseudo-R^2: 0.172
